# Supplementary material for: Building an understanding of Ethnic minority people’s Service Use Relating to Emergency care for injuries: the BE SURE study protocol
Source: BMJ Open. 2023 Apr 25;13(4):e069596. doi: 10.1136/bmjopen-2022-069596 (PMC10151843; doi:10.1136/bmjopen-2022-069596)
Supplement: Supplementary data [file bmjopen-2022-069596supp001.pdf]

BE SURE – Scoping Review Database Search Strategy

Databases Searched: EBSCO = CINAHL, MEDLINE AND PsycInfo; SCOPUS and COCHRANE

Limits applied – Date: January 2010 – May 2022; Human (only applied in EBSCO)

Search terms 1-5 combined with AND

1. **Population**:

(Ethnicit* OR Race OR “BAME” OR “Black, Asian and minorit* ethnic*” OR “Black, Asian & minorit* ethnic*” OR Immigrant* OR Migrant* OR “Asylum seeker*” OR Refugee* OR Multicultural OR Language OR English OR Ethnic OR Religion OR Culture OR Latin*o OR Latina OR Indigenous OR Aboriginal OR Native OR “First Nation*” OR “Afro-Caribbean*” OR "African American*” OR Gyps* OR Roma OR “Hispanic American”)

2. **Emergency Service:**

(“emergency service*” OR ambulance* OR “Emergency department*” OR “Accident and emergency department*” OR “Accident & emergency department*” OR A&E OR “emergency room*” OR “emergency care*” OR “999 calls and admission*” OR “Emergency health service*” OR 999 OR 911 OR “Ambulance service” OR “Ambulance trust” OR “Emergency medical service*” OR “EMS” OR “Patient transport” OR “control room”)

3. **Patient Category:**

(Patient OR Inpatient OR Outpatient OR “In-Patient” OR “Out-Patient”)

4. **Clinical care:**

(“Quality of care” OR Pain OR “Pain management” OR “length of hospital stay*” OR disability OR “Process of care” OR “Repeat attendance*” OR “Mortality” OR Treatment OR “Emergency treatment” OR “Clinical care” OR Rehabilitation)

5. **Injury:**

(Injur* OR assault* OR “Domestic violence” OR “Intimate partner violence” OR “self-harm” OR suicide OR accident* OR Violence)

Baghdadi F, *et al. BMJ Open* 2024; 13:e069596. doi: 10.1136/bmjopen-2022-069596
