# Supplementary material for: Building an understanding of Ethnic minority people’s Service Use Relating to Emergency care for injuries: the BE SURE study protocol
Source: BMJ Open. 2023 Apr 25;13(4):e069596. doi: 10.1136/bmjopen-2022-069596 (PMC10151843; doi:10.1136/bmjopen-2022-069596)
Supplement: Supplementary data [file bmjopen-2022-069596supp002.pdf]

BE SURE Interview Guide

Interviews with people identifying from an ethnic minority

Aim: to give people an opportunity to provide their own narrative about their circumstances, routes to seeking care, injury experiences (personal or family), views regarding their need, service use, care received, and post injury impact on physical and mental health including terminology (labels) used.

Guide

We have contacted you because you indicated on our survey that you wanted to participate in an interview to talk about your experience and journey of care on [*insert date*]. We will focus on that experience in this interview, but we will also ask other questions about your general experience of any other times in the past when you had an injury, and your use of ambulance and emergency services.

1. Can you tell me what happened on that day?
   a) Injury to self or other?
   b) Nature of injury or incident how, what, when it happened? Who else was there?
   c) What help did ambulance and emergency care on this occasion?

2. Why did you make the above choice?

3. What was the experience like calling 999?

   E.g. Prompts for their journey of care – calling 999, talking to someone on the phone, waiting, ambulance attending, ambulance to the hospital, transfer, receiving care at the hospital, aftercare - ask about feelings of being discriminated against at each stage and what type of, based on, and how discrimination – language, ethnicity, religion, gender, sexuality, lass, geography, etc.

4. What was the experience like in the emergency department?

BE SURE Interview Guide

E.g. Prompts for their **journey of care** – triage, waiting, receiving care at the hospital, aftercare - ask about feelings of being discriminated against at each stage and what type of, based on, and how discrimination – language, ethnicity, religion, gender, sexuality, class, geography, etc.

5. Have you had any other experience of getting health care after an injury?

a) How did that time compare with the time we've been talking about?

6. If you were injured would you call 999 and/or go to the emergency department again?

a) What would you think about when you decided what to do?

b) Who is the emergency department for?

7. Can you think of any other ways to get help after an injury?

a) Prompts: NHS111, pharmacy, GP, family/friends

Baghdadi F, *et al. BMJ Open* 2024; 13:e069596. doi: 10.1136/bmjopen-2022-069596
