# Supplementary material for: Building an understanding of Ethnic minority people’s Service Use Relating to Emergency care for injuries: the BE SURE study protocol
Source: BMJ Open. 2023 Apr 25;13(4):e069596. doi: 10.1136/bmjopen-2022-069596 (PMC10151843; doi:10.1136/bmjopen-2022-069596)
Supplement: Supplementary data [file bmjopen-2022-069596supp003.pdf]

BE SURE

NIHR | National Institute for Health Research

Swansea University Prifysgol Abertawe
Medical School Ysgol Feddygaeth

<<Study ID>>

A. Your Details

1. What is your gender ☐ Female ☐ Male ☐ Other ☐ Prefer not to say

2. What is your age in years? ☐

3. What ethnicity do you identify as?

- ☐ White British
- ☐ White Irish
- ☐ Other White
- ☐ White/Black Caribbean
- ☐ White/Black African
- ☐ White/Asian
- ☐ Other mixed
- ☐ Indian
- ☐ Pakistani
- ☐ Bangladeshi
- ☐ Other Asian
- ☐ Caribbean
- ☐ African
- ☐ Other Black
- ☐ Chinese
- ☐ Other ethnic group?

Please Specify ☐

4. What is your preferred language?

- ☐ English
- ☐ Scots
- ☐ Polish
- ☐ Punjabi
- ☐ Urdu
- ☐ Bengali
- ☐ Gujarati
- ☐ Arabic
- ☐ French
- ☐ Mandarin
- ☐ Cantonese
- ☐ Spanish
- ☐ Portuguese
- ☐ Tamil
- ☐ Turkish
- ☐ Slavak
- ☐ Somali
- ☐ Lithuanian
- ☐ German
- ☐ Persian/Farsi
- ☐ Tagalog/Filipino
- ☐ Romanian
- ☐ Other

☐

BE SURE NIHR | National Institute for Health Research Swansea University Prifysgol Abertawe Medical School Ysgol Feddygaeth

<<Study ID>>

5. What do you feel is your level of English?

Please tick all that apply.

5.1. Reading.

- ☐ I can read English well
- ☐ I can read a little English
- ☐ I cannot read English

5.2. Speaking.

- ☐ I can speak English well
- ☐ I can speak a little English
- ☐ I cannot speak English

5.3. Holding a conversation.

- ☐ I can talk in English with a health professional (i.e. doctor, nurse, or paramedic)
- ☐ I find it difficult to talk in English with a health professional
- ☐ I cannot talk in English with a health professional

6. How long have you lived in the UK?

- ☐ My whole life, go to question 7
- ☐ 1-5 years
- ☐ More than 5 years
- ☐ Less than 1 year

6.1. What country were you living in before settling in the UK?

☐

B. Your knowledge of healthcare services

In this section we want to understand what you know about the different emergency healthcare services that are available.

7. Do you know healthcare care is free in the United Kingdom?

- ☐ Yes
- ☐ No

IRAS 305391 BE SURE Questionnaire

Version 1.3 26/01/2023

BE SURE | NIHR | National Institute for Health Research | Swansea University Prifysgol Abertawe Medical School Ysgol Feddygaeth

<<Study ID>>

8. Do you know 999 ambulance service care is free in the United Kingdom?

☐ Yes ☐ No ☐ Not Sure

9. Do you know Emergency Department care is free in the United Kingdom?

☐ Yes ☐ No ☐ Not Sure

10. Have you heard of the following services?

	Not heard of the service	Heard of the service but don't know how to contact	Have heard of and would know how to access the service
NHS 111/NHS Direct telephone advice			
Minor Injuries Unit			
999 ambulance service			
Emergency Department (A&E)			

11. Did you know that the NHS should provide you with an interpreter if you need one?

☐ Yes ☐ No ☐ Not Sure

C. Who you contact when hurt

12. How often have you called the 999 ambulance service in the last 12 months for an injury?

☐ 0 ☐ 1-2 ☐ 2-5 ☐ 5+

BE SURE | NIHR National Institute for Health Research | Swansea University Prifysgol Abertawe Medical School Ysgol Feddygaeth

<<Study ID>>

13. How often have you visited an Emergency Department in the last 12 months for an injury?

☐ 0 ☐ 1-2 ☐ 2-5 ☐ 5+

D. Your experience of care

In this section we want to understand your experience of healthcare provided by the 999 ambulance service, Emergency Department, or both on the date on the cover letter of this questionnaire. You only need to answer questions that are relevant to your experience.

Calling 999 ambulance service

14. Did you make the 999 ambulance service call?

☐ Yes ☐ No if no – go to Q.27, Page 5.

15. Do you feel that the 999 ambulance service call taker listened to you?

☐ Yes, completely ☐ Yes, somewhat ☐ No ☐ Don't know

16. Did you think that the 999 ambulance service call taker asked questions relevant to your problem?

☐ Yes, completely ☐ Yes, somewhat ☐ No ☐ Don't know

17. Did you feel reassured when speaking to the 999 ambulance service call taker?

☐ Yes, completely ☐ Yes, somewhat ☐ No ☐ Don't know

BE SURE | NIHR National Institute for Health Research | Swansea University Prifysgol Abertawe Medical School Ysgol Feddygaeth

<<Study ID>>

18. Did the 999 ambulance service call taker tell you an ambulance would be sent to you?

- ☐ Yes
- ☐ No

If no, what did the call taker advise? Tick all that apply and go to Q.27, Page 7

- ☐ Manage the problem at home yourself
- ☐ Go to your GP
- ☐ Go to the Pharmacist
- ☐ Call NHS 111
- ☐ Call back if the problem gets worse

19. If you needed help, did the 999 ambulance service call taker tell you how long you could expect to wait for help to arrive?

- ☐ Yes, but wait was shorter
- ☐ Yes, and that was about right
- ☐ Yes, but wait was longer
- ☐ No
- ☐ Don't know

20. How would you rate your experience of using 999 ambulance service telephone service on this occasion?

- ☐ Very good
- ☐ Good
- ☐ Fair
- ☐ Poor
- ☐ Very Poor

When the Ambulance Arrived

21. Did the paramedics or ambulance workers you saw explain what they were doing in a way you could understand?

- ☐ Yes, completely
- ☐ Yes, somewhat
- ☐ No
- ☐ Don't know

Baghdadi F, *et al. BMJ Open* 2024; 13:e069596. doi: 10.1136/bmjopen-2022-069596

BE SURE NIHR | National Institute for Health Research Swansea University Prifysgol Abertawe Medical School Ysgol Feddygaeth

<<Study ID>>

22. Did the paramedics or ambulance workers explain what was wrong with you in a way you could understand?

☐ Yes, completely ☐ Yes, somewhat ☐ No ☐ Don't know

23. If you were in pain, did the paramedics or ambulance workers do all they could to help?

☐ Yes, definitely ☐ Yes, somewhat ☐ No ☐ I was not in any pain

24. Once the paramedics or ambulance workers completed their work, did they tell you what to do if you continued to feel unwell or if your condition worsened?

☐ Yes, they told me to call the ambulance service back ☐ Yes, they told me to call my GP ☐ Yes, they told me to call another health care professional ☐ Yes, they gave me an information leaflet ☐ No, they did not provide any advice if I continued to feel unwell ☐ No advice was wanted/ needed ☐ Don't know

25. Overall, how well do you think you were looked after by the paramedics or ambulance workers?

☐ Very well ☐ Fairly well ☐ Not very well ☐ Not at all well

26. Did you travel to the Emergency Department in the ambulance?

☐ Yes ☐ No – if no, got to Q32, Page 8.

Journey to Emergency Department

27. Was it your decision to go to the Emergency Department?

BE SURE | NIHR National Institute for Health Research | Swansea University Prifysgol Abertawe Medical School Ysgol Feddygaeth

<<**Study ID**>>

☐ Yes ☐ No ☐ Not sure

28. Was the length of time the ambulance workers spent with you before taking you to hospital or elsewhere what you expected?

☐ Yes ☐ No, it was too long ☐ No, it was too short ☐ Don't know

29. Did the ambulance workers talk to you during the journey? (Please tick all that apply)

☐ Yes, they told me what was happening

☐ Yes, they asked me about how I was feeling

☐ Yes, they talked to me about what would happen when we got to the hospital or elsewhere

☐ Yes, they talked about something else

☐ No, they didn't talk to me

30. How well do you feel you understood what the paramedics or ambulance workers were saying to you during your journey?

☐ Very well ☐ Well ☐ Neutral ☐ Not well ☐ Not at all

31. When you arrived at the hospital did the paramedics or ambulance workers wait with you until you were seen by a doctor or nurse?

☐ Yes ☐ No ☐ Can't remember

Go to Q.41, Page 9.

BE SURE | NIHR | National Institute for Health Research | Swansea University Prifysgol Abertawe Medical School Ysgol Feddygaeth

<<**Study ID**>>

Arriving at the Emergency Department yourself

Please complete this section only if you travelled to the Emergency Department without an ambulance; if an ambulance brought you to the Emergency Department - go to Q.41, Page 9.

32. Who did you go to the Emergency Department with?

☐ Myself ☐ Family member ☐ Other (specify)

☐ Friend ☐ Care worker

☐ Neighbour ☐ Health professional

33. When you arrived at the Emergency Department, was it clear to you where to register?

☐ Yes, definitely ☐ Yes, somewhat ☐ No, this was not needed ☐ No, this would have helped ☐ Can't remember

34. Did anyone help to explain your problem to the person who registered you?

☐ Friend ☐ Care Worker ☐ Not Applicable

☐ Neighbour ☐ Family ☐ Other (please specify)

☐ Another patient in Emergency Department

35. Did the person who registered you understand your problem?

☐ Yes, completely ☐ Yes, somewhat ☐ No ☐ Don't know ☐ Not Applicable

36. After your registration, did you have to wait to be seen again?

☐ Yes ☐ No ☐ Can't remember (If no or can't remember, go to Q.41, Page 9.)

37. Was the length of time you waited to be seen again what you expected?

☐ I did not have to wait at all ☐ The wait was shorter than expected ☐ The wait was about as long as expected ☐ The wait was longer than expected ☐ Don't Know

BE SURE | NIHR | National Institute for Health Research | Swansea University Prifysgol Abertawe Medical School Ysgol Feddygaeth

<<Study ID>>

38. While you were waiting, did someone tell you what was happening?

☐ Yes, definitely ☐ Yes, somewhat ☐ No, this was not needed ☐ No, this would have helped ☐ Can't remember

39. While you were waiting, did someone tell you what to do?

☐ Yes, definitely ☐ Yes, somewhat ☐ No, this was not needed ☐ No, this would have helped ☐ Can't remember

40. While you were waiting, did you get all the things you needed (like medicine, food, drink and toilets)?

☐ Yes, definitely ☐ Yes, somewhat ☐ No ☐ I did not need anything ☐ Don't know

Receiving Care

41. Could you understand when Emergency Department staff explained…

41.1. What they were doing?

☐ Yes, completely ☐ Yes, somewhat ☐ No ☐ Don't know

41.2. What was wrong with you?

☐ Yes, completely ☐ Yes, somewhat ☐ No ☐ Don't know

42. If you were in pain, did Emergency Department staff do all they could to help your pain?

☐ Yes, definitely ☐ Yes, somewhat ☐ No ☐ I was not in pain ☐ Don't know

BE SURE | NIHR | National Institute for Health Research | Swansea University Prifysgol Abertawe Medical School Ysgol Feddygaeth

<<Study ID>>

43. Were you given enough privacy when being treated and examined?

☐ Yes, definitely ☐ Yes, somewhat ☐ No ☐ I did not want privacy ☐ Don't know

44. After your attendance at the Emergency Department, what happened?

☐ I was admitted to hospital

☐ I was told to go home

☐ I was told to go see another health care worker (please specify)

☐ I discharged myself and went..., (please specify where)

45. Did anyone at the Emergency Department tell you when you could restart your usual activities?

☐ Yes definitely ☐ Yes somewhat ☐ No ☐ This was not necessary ☐ Don't know

46. Did anyone at the Emergency Department tell you how to provide self-care at home after your hospital care?

☐ Yes definitely ☐ Yes somewhat ☐ No ☐ This was not necessary ☐ Don't know

47. Did anyone at the Emergency Department tell you what to do or who to contact if you were still worried about anything?

☐ Yes ☐ No ☐ Don't know

Baghdadi F, et al. BMJ Open 2024; 13:e069596. doi: 10.1136/bmjopen-2022-069596

BE SURE | NIHR | National Institute for Health Research | Swansea University Prifysgol Abertawe Medical School Ysgol Feddygaeth

<<Study ID>>

E. Satisfaction with Care

Satisfaction with 999 Ambulance Service

If you travelled to the Emergency Department without an ambulance - go to Q.50, Page 13.

48. Please read the statements below and place an **X** in the box that reflects your agreement with each.

	Not applicable	Strongly agree	Agree	Neither agree nor disagree	Disagree	Strongly disagree
The ambulance crew who attended me listened carefully to my problem						
I think the crew were polite						
I got the advice I needed						
I was reassured by the advice given to me						
I was satisfied with the explanation I was given						
I was given advice about when to get more help						
I was satisfied with the ambulance crew						
I was made to feel I was wasting the crew's time						

BE SURE | NIHR National Institute for Health Research | Swansea University Prifysgol Abertawe Medical School Ysgol Feddygaeth

<<Study ID>>

49. If the same health problem arose again, would you call an ambulance to help you with your problem?

☐ Yes ☐ No ☐ Don't know

If no, who would you contact for advice or help, or would you look after yourself?

Tick any that apply.

☐ I would go to ED myself	☐ Careline	☐ District Nurse
☐ I would look after myself	☐ GP	☐ Social Worker
☐ NHS Direct/NHS111	☐ Mental Health Team	☐ Other – please specify

BE SURE

NIHR | National Institute for Health Research

Swansea University Prifysgol Abertawe
Medical School Ysgol Feddygaeth

<<Study ID>>

Satisfaction with Emergency Department (A&E)

50. Please read statements below and place an X in the box that reflects your agreement with each.

	Not applicable	Strongly agree	Agree	Neither agree nor disagree	Disagree	Strongly disagree
The Emergency Department staff who supported me listened carefully to my problem						
I think the staff were polite						
I got the amount of advice I needed						
I was reassured by the advice I received						
I was satisfied with the explanation I received						
I was given advice about when to get more help						
I was generally satisfied with the Emergency Department staff						
I was made to feel I was wasting the worker's time						

BE SURE | NIHR National Institute for Health Research | Swansea University Prifysgol Abertawe Medical School Ysgol Feddygaeth

<<Study ID>>

51. If the same health problem arose again, would you go to the Emergency Department to help you with your problem?

☐ Yes ☐ No ☐ Don't know

If no, who would you contact for advice or help, or would you treat/look after yourself?

Tick any that apply.

☐ I would call 999	☐ GP	☐ Social Worker
☐ I would look after myself	☐ Mental Health Team	☐ Careline
☐ NHS Direct/NHS 111	☐ District Nurse	☐ Other – please specify

F. Your health and wellbeing

This section asks for your views about your health and how well you are able to do your usual activities. **Answer each question by choosing just one answer**. If you are unsure how to answer a question, please give the best answer you can.

1. **In general, would you say your health is:**

☐	☐	☐	☐	☐
Excellent	Very Good	Good	Fair	Poor

The following questions are about activities you might do during a typical day. Does your health now limit you in these activities? If so, how much?

	YES, limited a lot	YES, limited a little	NO, not limited at all
2. **Moderate activities such as** moving table, pushing vacuum cleaner, bowling, or playing golf.	☐	☐	☐
3. Climbing **several** flights of stairs.	☐	☐	☐

Baghdadi F, *et al. BMJ Open* 2024; 13:e069596. doi: 10.1136/bmjopen-2022-069596

BE SURE NIHR | National Institute for Health Research Swansea University Prifysgol Abertawe Medical School Ysgol Feddygaeth

<<Study ID>>

During the past 4 weeks, have you had any of the following problems with your work or other daily activities as a result of your physical health?

	YES	NO
4. **Accomplished less** than you would like.	☐	☐
5. Were limited in the **kind** of work or other activities.	☐	☐

During the past 4 weeks, have you had any of the following problems with your work or other daily activities as a result of any emotional problems (like feeling anxious or depressed)?

	YES	NO
6. **Accomplished less** than you would like.	☐	☐
7. Did work or activities **less carefully than usual**.	☐	☐

8. **During the past 4 weeks, how much did pain interfere with your normal work (including work outside the home and housework)?**

☐	☐	☐	☐	☐
Not at all	A little	Moderately	A lot	Extremely

Baghdadi F, *et al. BMJ Open* 2024; 13:e069596. doi: 10.1136/bmjopen-2022-069596

BE SURE | NIHR | National Institute for Health Research | Swansea University Prifysgol Abertawe Medical School Ysgol Feddygaeth

<<Study ID>>

These questions are about how you have been feeling during the past 4 weeks.
For each question, please give the answer that comes closest to the way you have been feeling.
How much of the time during the past 4 weeks…

	All of the time	Most of the time	A good bit of the time	Some of the time	A little bit of the time	None of the time
9. Have you felt calm and peaceful?	☐	☐	☐	☐	☐	☐
10. Did you have a lot of energy?	☐	☐	☐	☐	☐	☐
11. Have you felt down-hearted and blue?	☐	☐	☐	☐	☐	☐

12. **During the past 4 weeks, how much of the time has your physical health or emotional problems interfered with your social activities (like visiting friends, relatives, etc.)?**

☐ All of the time ☐ Most of the time ☐ Some of the time ☐ A little of the time ☐ None of the time

G. Final questions and thank you

52. Please enter today's date. [ ]

53. Did anyone help you to complete this questionnaire (family, friend, support worker, or researcher)?

☐ Yes ☐ No

If yes, please describe your relationship with your helper.

[ ]

IRAS 305391 BE SURE Questionnaire

Version 1.3 26/01/2023

BE SURE | NIHR National Institute for Health Research | Swansea University Prifysgol Abertawe Medical School Ysgol Feddygaeth

<<Study ID>>

We would like to link your questionnaire to your routine NHS information anonymously.

Place an **X** in the box if you **DO NOT** agree for us to link your questionnaire answers to your NHS information. ☐

54. We would like to invite you to take part in a one-to-one interview by telephone or in person to talk about the experience you described in this questionnaire. Would you be interested in talking to us about your experience of care?

☐ Yes ☐ No

If yes, please provide your contact details and we will arrange a convenient time and date for the interview.

Name	
Phone	
Email	

If you would like to receive a £10 High Street voucher for completing this questionnaire, please provide your full name and email address below. If you do not have an email address, please provide your full home address including post code.

Name	
Email	
Address and post code	

Thank you for your helping us with this study.
