# Supplementary material for: Building an understanding of Ethnic minority people’s Service Use Relating to Emergency care for injuries: the BE SURE study protocol
Source: BMJ Open. 2023 Apr 25;13(4):e069596. doi: 10.1136/bmjopen-2022-069596 (PMC10151843; doi:10.1136/bmjopen-2022-069596)
Supplement: Author's manuscript [file bmjopen-2022-069596.draft_revisions.pdf]

BMJ Open

BMJ Open is committed to open peer review. As part of this commitment we make the peer review history of every article we publish publicly available.

When an article is published we post the peer reviewers' comments and the authors' responses online. We also post the versions of the paper that were used during peer review. These are the versions that the peer review comments apply to.

The versions of the paper that follow are the versions that were submitted during the peer review process. They are not the versions of record or the final published versions. They should not be cited or distributed as the published version of this manuscript.

BMJ Open is an open access journal and the full, final, typeset and author-corrected version of record of the manuscript is available on our site with no access controls, subscription charges or pay-per-view fees (http://bmjopen.bmj.com).

If you have any questions on BMJ Open's open peer review process please email info.bmjopen@bmj.com

BMJ Open

Building an understanding of Ethnic minority people's Service Use Relating to Emergency care for injuries: the BE SURE study protocol

Journal:	*BMJ Open*
Manuscript ID	bmjopen-2022-069596
Article Type:	Protocol
Date Submitted by the Author:	27-Oct-2022

Complete List of Authors:	Baghdadi, Fadi; Swansea University, Medical School<br>Bell, Fiona; Yorkshire Ambulance Service NHS Trust<br>Evans, Bridie; Swansea University, Swansea University Medical School Medicine<br>Goodacre, Steve; University of Sheffield, Medical Care Research Unit<br>Hettiarachchi, Thanuja; Swansea University, Public contributor, c/o Medical School<br>John, Ann; Swansea University, Medical sChool<br>John, Paul; Scottish Ambulance Service<br>Khanom, Ashrafunnesa; Swansea University, School of Medicine<br>Lyons, Ronan; University of Wales Swansea, Swansea Clinical School<br>Siriwardena, Aloysius; University of Lincoln, Community and Health Research Unit<br>Porter, Alison; University of Swansea, College of Medicine<br>Russell, Ian; Swansea University, Medicine<br>Safari, Solmaz; Swansea University, Public contributor, c/o Medical School<br>Watkins, Alan; Swansea University, College of Medicine<br>Williams, Julia; University of Hertfordshire<br>Snooks, Helen; Swansea University, Medicine

Keywords:	ACCIDENT & EMERGENCY MEDICINE, PUBLIC HEALTH, QUALITATIVE RESEARCH

SCHOLARONE™
Manuscripts

Title Page

Building an understanding of Ethnic minority people's Service Use Relating to Emergency care for injuries: the BE SURE study protocol

Fadi Baghdadi, Medical School, Swansea University, Swansea, United Kingdom

Fiona Bell, Yorkshire Ambulance Service NHS Trust, Sheffield, United Kingdom

Bridie Angela Evans, Medical School, Swansea University, Swansea, United Kingdom

Steve Goodacre, School of Health and Related Research, University of Sheffield, Sheffield, United Kingdom

Thanuja Hettiarachchi, Public contributor, c/o Medical School, Swansea University, Swansea, United Kingdom

Ann John, Medical School, Swansea University, Swansea, United Kingdom

John Paul Anthony, Research and Innovation Hub, Scottish Ambulance Service, Edinburgh, United Kingdom

Ashrafunnesa Khanom, Medical School, Swansea University, Swansea, United Kingdom

Ronan Lyons, Medical School, Swansea University, Swansea, United Kingdom

Aloysius Niroshan Siriwardena AN, School of Health & Social Care, University of Lincoln, Lincoln, United Kingdom

Alison Porter, Medical School, Swansea University, Swansea, United Kingdom

Ian Russell, Medical School, Swansea University, Swansea, United Kingdom

Solmaz Safari, Public contributor, c/o Medical School, Swansea University, Swansea, United Kingdom

Alan Watkins, Medical School, Swansea University, Swansea, United Kingdom

Julia Williams, University of Hertfordshire, Hertfordshire, United Kingdom

Helen Snooks, Medical School, Swansea University, Swansea, United Kingdom

Corresponding author: Fadi Baghdadi

Address: Office – 205, Second Floor, Institute of Life Science 2, Singleton Campus, Sketty, Swansea, SA2 8PP, United Kingdom.

Email: fadi.baghdadi@swansea.ac.uk

Phone number: +44 1792 513409

Word Count = 3,896

For peer review only - http://bmjopen.bmj.com/site/about/guidelines.xhtml

Building an understanding of Ethnic minority people's Service Use Relating to Emergency care for injuries: the BE SURE study protocol

ABSTRACT

Introduction

Injuries are a major public health problem which can lead to disability or death. However, little is known about the incidence, presentation, management and outcomes of emergency care for patients with injuries among people from ethnic minorities in the United Kingdom. The aim of this study is to investigate what may differ for people from ethnic minorities compared to white British people when presenting with injury to ambulance and emergency departments (EDs).

Methods and Analysis

This mixed methods study covers eight services, four ambulance services (three in England and one in Scotland) and four hospital EDs, located within each ambulance service. The study has five work packages (WP): (WP1) scoping review comparing mortality by ethnicity of people presenting with injury to emergency services; (WP2) retrospective analysis of linked NHS routine data from patients who present to ambulances or EDs with injury over five years (2016-2021); (WP3) postal questionnaire survey of 2,000 patients (1,000 patients from ethnic minorities and 1,000 white British patients) who present with injury to ambulances or EDs including self-reported outcomes (measured by Quality of Care Monitor and Health Related Quality of Life measured by SF-12); (WP4) qualitative interviews with patients from ethnic minorities (n = 40) and focus groups – four with asylum seekers and refugees and four with care providers; and (WP5) a synthesis of quantitative and qualitative findings.

Ethics and Dissemination

This study received a favourable opinion by the Wales Research Ethics Committee (305391). The Health Research Authority has approved the study and, on advice from the Confidentiality Advisory Group, has supported the use of confidential patient information without consent for anonymised data. Results will be shared with Ambulance and ED services, government bodies and third sectors organisations through direct communications summarising scientific conference proceedings and publications.

For peer review only - http://bmjopen.bmj.com/site/about/guidelines.xhtml

Strengths and limitations of this study

Strengths:

- Mixed quantitative and qualitative methods will ensure representativeness and depth in addressing our research questions
- The study will use peer researchers from ethnic minorities recruited by local third sector organisations to support people to complete questionnaires to ensure local buy in and to achieve as high a response rate to the survey as possible
- Routine linked data allows the inclusion of a large number of patients and attendances over a 5-year period, producing a comprehensible epidemiological picture

Limitations:

- Coding of ethnicity may be inaccurately recorded or incomplete in routine health records which may mask heterogeneity within each group
- Response rates to questionnaire surveys may be low and differ between cohorts, introducing potential bias to findings

Keywords

Emergency Medical Services

Wounds and Injuries

Electronic Health Records

Medical Record Linkage

Minority Health

Ethnicity in Health Services

For peer review only

INTRODUCTION

Injuries cause 5 million deaths worldwide each year and many more people are left with disability [1]. In the United Kingdom (UK) around six million Emergency Department (ED) visits result from accidental injuries each year [2] and over 14,000 of these injuries result in death [3].

A founding and sustained principle of the National Health Service (NHS) is that there should be equity of access and treatment for all [4]. However, disparities in access to health care and health outcomes for people from ethnic minorities compared to white British people have been regularly reported[5]. Future changes in the delivery of NHS care as proposed in the NHS Long Term Plan [6] may deepen inequalities, as people with urgent care needs including minor injuries are redirected towards NHS 111 (telephone service) and GP led Urgent Treatment Centres. Following COVID-19, further initiatives have been trialled to control immediate access to emergency care[7]. However, there are concerns that people from ethnic minorities are more likely to make greater use of emergency healthcare, reflecting difficulties in accessing primary care [8]. In the Health Experiences of Asylum Seekers and Refugees (HEAR) study, [9] 77% of survey respondents knew about the 999 service, but only 28% were aware of the Out of Hours General Practitioner (GP) service. Research across Europe [10] reports a rise in migrants' and asylum seekers' use of emergency services. High use has been associated with language barriers, social deprivation, poor access to primary care [10], delayed or restricted access to secondary healthcare [11], or people falling through gaps between other services (such as community mental health services) [12].

People from ethnic minorities across Europe, North America and Oceania have been widely reported to have differences in access, experiences, and outcomes when presenting to

For peer review only - http://bmjopen.bmj.com/site/about/guidelines.xhtml

emergency services. People from ethnic minorities who present with injuries have different experiences in relation to pain management [3], length of hospital stay [14], quality of care [15], disability [16], repeat attendance [17], and mortality [18]. They also have increased risk of certain injury presentations including; gunshot injuries [19], long bone fractures [20], head injuries [21], alcohol related injury [22], workplace injury [23], assaults [24], self-harm and attempted suicide [25] and Female Genital Mutilation (FGM) [26]. However, people from ethnic minorities have lower prevalence of other injuries including: falls among the elderly and road traffic injuries [27], fire injuries [28] and partner violence [29].

While death and morbidity rates due to injury are higher in some ethnic minority populations in the UK [30], there remains a gap in evidence on their experiences of emergency services. This is partly due to a lack of focus or priority on this area of inequality until recently [31]. Firstly, there is a weakness in routine information systems, where ethnicity data are often poorly recorded, particularly in emergency prehospital care settings [32]. Secondly, the preferred language of patients from ethnic minorities are not recorded in routine health data, nor are differences in culture and language adequately accommodated for in emergency services, with a scarcity of government funded interpreters [33], public health campaigns [34] and allied health services [35]. There is considerable scope for taking a more analytical approach to studying injury presentation and differences in emergency care among people from ethnic minorities in the UK that will inform policy and practice and help to reduce future disparities and burden of injury, mortality and disability.

Study Aim

To describe disparities in injury presentation, processes of care and outcomes between people from ethnic minorities and white British people when they contact emergency health services for injury.

For peer review only

Objectives

We will:

1) Describe the published literature reporting all-cause mortality of people presenting with injury to emergency services by ethnicity

2) Describe the quality (completeness, consistency) of ethnicity data in routine emergency healthcare datasets

3) Compare between people from ethnic minorities and white British people: injury type, severity, care delivered, outcomes, beliefs and experiences when they contact emergency health services for injuries

4) Explore with people from ethnic minorities, including refugees and asylum seekers: knowledge of service availability, factors which deter or encourage them to seek help, experiences of emergency healthcare for injuries

5) Explore emergency healthcare providers' experiences of delivering care to people from ethnic minorities presenting with injury

6) Synthesise quantitative and qualitative findings to:

    a. Help policy makers and care providers to develop and implement interventions to promote accessibility of services for injury in ethnic minorities populations

    b. Enable ambulance service and EDs to improve care and outcomes for people in these populations with injuries

    c. Inform injury surveillance resources to include ethnicity in their reporting of injury

METHODS

Setting

We will conduct this study in the catchment area of one receiving hospital ED within each of four ambulance services. We selected sites where an established electronic patient data capture system was in place in the ambulance service. The participating ambulance services will provide linkable electronic datasets including ethnicity codes, which are available in

For peer review only - http://bmjopen.bmj.com/site/about/guidelines.xhtml

For peer review only

approximately 70% of patient records [32]. We hope to retrieve 80% of centrally held ED records with ethnicity codes [36].

Third sector organisations who provide support in relation to health care for people from ethnic minorities at each site will help connect researchers with the local population. They will promote the study across their networks to encourage people to respond to postal survey questionnaires and provide support with recruiting and managing peer researchers to support sampled patients to complete the questionnaires.

Study Design

We will use a convergent model of data collection where the quantitative and qualitative methods are conducted in parallel, and results are merged during the interpretation stage [37]. We will examine disparities in experiences, injury presentation, processes of care and outcomes as both the product of the individual patients' decisions and actions [38], and the organisations decisions, actions and attitudes [39]. As shown in Figure 1, in our Work Packages (WP) we will:

- WP1) Conduct a scoping review of existing literature.
- WP2) Retrieve and analyse retrospective linked NHS routine data over a five-year period (2016-2021) related to ambulance and ED contacts by patients from ethnic minorities and white British patients for injury to compare demographics, casemix, processes and outcomes of care.
- WP3) Conduct a questionnaire survey with samples of people from ethnic minorities and white British people who contacted the ambulance service or attended ED for an injury within a specified recent period of up to 6 months to compare self-reported experiences, satisfaction and health related quality of life.

For peer review only - http://bmjopen.bmj.com/site/about/guidelines.xhtml

WP4) Conduct in-depth interviews in each site with people from ethnic minorities who consent to be contacted for an interview in their completed questionnaires and conduct one focus group with refugees and/or asylum seekers at each site and one focus group with stakeholders at each site: e.g., ED clinicians, paramedics, GPs and other primary care staff, social services staff, and third sector support workers.

WP5) Synthesise our findings from quantitative and qualitative data to generate key messages and implications for policy and service delivery.

Figure 1. Study design and participant recruitment flowchart

WP 1: Scoping review

We will undertake a scoping review following the Joanna Briggs Institute (JBI) methodology [40]. We will refer to the Preferred Reporting Items for Systematic Reviews and Meta-analysis Extension for Scoping Reviews (PRISMA - ScR) [41] and JBI reporting checklists to developing a scoping review protocol [42]. The scoping review will aim to describe the published literature reporting cases of mortality by race or ethnicity of adults presenting with injury to emergency services.

We will identify papers from database searches which will be screened independently by title, abstract and full paper following a protocol by at least two reviewers from the research team. We will include studies that report all cases of mortality by race or ethnicity of adults presenting to emergency services for injury. We will exclude studies involving people with no ethnicity record; no record of injury as the cause of emergency service use; no reporting of mortality by race and ethnicity and those reporting non-emergency care such as scheduled appointments, outpatient department services and elective surgeries. All discrepancies between reviewers will be resolved by a third reviewer.

For peer review only - http://bmjopen.bmj.com/site/about/guidelines.xhtml

WP 2: Epidemiology of injury presentation, care delivery and outcomes using anonymised linked routine data

We will link routine ambulance service data between August 2016 and July 2021 related to patients presenting with injury within the ambulance service catchment area of each participating ED to centrally held ED, inpatient, outpatient and Office of National Statistics (ONS) datasets (using NHS Digital in England and eDRIs in Scotland) [43]. Individual-level prehospital data on calls made for injury will be retrieved by each ambulance service from its Computer Aided Dispatch (CAD) and Patient Clinical Record (PCR) systems; these data are currently unavailable in NHS Digital or Electronic Data Research and Innovation Service (eDRIS). Clinical data will include ethnicity; condition code; job cycle time (from first 999 call for the incident to time ambulance reported free to respond to next 999 call); medications given and disposition (conveyed to hospital, treated without conveyance). We will link this data using a study-specific Identity (ID) to patient identifiable data held in separate files – the "split file" method [44] – and uploaded to NHS Digital or eDRIS by each site. We will then use patient identifiable data within NHS Digital or eDRIS to create Anonymised Linkage Fields (ALFs) and retrieve routinely recorded outcomes for these patients (Figures 2).

Figure 2. WP2 – Data flow for routine linked data epidemiology of injury presentation, care delivery and outcomes

We will also retrieve routine data on ED attendances for injury from participating EDs for the same period from NHS Digital / eDRIS. We will then retrieve anonymised linked routine health outcomes for six months after index presentation with injury to ambulance services and EDs, to assess outcomes unless the person has specifically opted out [45]. We will request data related to: diagnoses; disposition from ambulance service and ED; length of stay at index episode in hospital; treatments received and discharge code; Intensive Care Unit (ICU) admissions and length of stay; further ED attendances and emergency admissions; and deaths up to six months.

For peer review only - http://bmjopen.bmj.com/site/about/guidelines.xhtml

We will partition the aggregated data into cohorts of patients from ethnic minorities, patients from the white British population and those for whom no ethnicity is recorded; with appropriate sub-groups identified using 2011 Census ethnicity categories [46]. We will include patients with multiple presentations or attendances with the first presentation or attendance as baseline, and data from subsequent presentations or attendances contributing to outcomes. We will hold a consultation workshop with stakeholders at the outset of the study to help clarify and define our study outcome measures.

We will compare patterns of presentation, processes of care and outcomes through cross-sectional analyses to investigate differences in:

- Demographics; geography and deprivation index; mechanism of injury; severity; injury type (accidental, non-accidental, assault, self-harm); casemix; route to care (direct, via 111 telephone advice service or via general practice)
- Treatments and investigations
- Potential safety incidents (e.g., hospital admission or death within 72 hours of discharge from 999 or ED care) following injury
- Immediate outcomes (at index event) including ambulance attendance, transportation to hospital, hospital admission, length of stay in hospital and ICU, death following injury
- 6-month outcomes (further ED or hospital attendances, length of stay in hospital and deaths) following injury

WP 3: Questionnaire survey

We will survey people from ethnic minorities and white British people who have presented with injury to one of the four ambulance services or nominated ED. Each of the four study sites will search through their routine ambulance service and ED records to identify patients

For peer review only - http://bmjopen.bmj.com/site/about/guidelines.xhtml

presenting with injury and coded as being from an ethnic minority, including patients who presented to the ambulance service but were not conveyed to hospital. They will also identify a similar sized cohort of patients identified as white British.

Each study site (comprising one ambulance service and one ED) will send out 500 postal questionnaires (n=2000 in total, 4 sites) to 250 patients from ethnic minorities and 250 white British patients. Before sending out questionnaires, the clinical care team will check death records to ensure that the person has not died, to avoid causing distress to their family. All recipients will be asked to return completed questionnaires in a prepaid envelope to Swansea University. A reminder letter will be sent after two weeks. Recipients will also have the option to complete the questionnaire online (via a secure approved platform) using a QR code, reducing the potential burden of having to return the questionnaire by post. Where patient contact numbers are available, the clinical care team will contact the patient by telephone one week after sending the questionnaire, to offer support to complete the questionnaire over the telephone, or to refer the participant to a local peer researcher to provide this support where consent is provided. The expected 800 analysable questionnaire responses will (using 90% power, 5% significance) enable us to detect differences in outcomes equivalent to a standardised statistical effect of ~0.23; this, in turn, corresponds to clinically meaningful differences in study outcomes (e.g., self-reported Health Related Quality of Life). We will offer all respondents a £10 voucher for completing the questionnaire [47]. All questionnaire data will be inputted and stored on secure Swansea University database.

We will base our survey questions on those used successfully in the HEAR survey,**Error! Bookmark not defined.** focusing on knowledge of services, beliefs, experiences of injury, expectations, and health-seeking behaviour. The questionnaire will also include standardised

For peer review only - http://bmjopen.bmj.com/site/about/guidelines.xhtml

questionnaires to measure satisfaction with care (Quality of Care Monitor) [48] and current health status (SF-12) [49]. The questionnaire will be translated into several languages and translated versions will be available on request.

We will recruit and train twelve community peer researchers [50] from ethnic minorities to support with the collection of questionnaire data. We will recruit and train peer researchers with support from our third sector partner organisations at each site. The peer researchers will work closely with the clinical care team in their localities who will refer respondents who request language support to the appropriate peer researcher with the patient's consent to help complete the questionnaire.

Identifiable data for all patients who are sampled to receive the questionnaire will be saved by participating services in a "split file" format and uploaded into NHS Digital or eDRIS (Figure 3). Questionnaire responses will be linked to clinical records from ED (HES and Emergency Care Data Set (ECDS) (England); Hospital Activity Statistics (HAS) and Ambulance & Emergency Datamart (A&ED) (Scotland) and hence to factors and covariates derived from these data sources.

Figure 3. WP3 – Data flow for questionnaire survey

WP 4: Qualitative interviews and focus groups

We will conduct a total of 40 interviews with patients from ethnic minorities (10 in each site, identified from survey respondents who provide their consent and contact details) and four focus groups with Asylum Seekers and Refugees (up to six participants per site, identified by third sector organisations).

For peer review only - http://bmjopen.bmj.com/site/about/guidelines.xhtml

We will purposively sample survey respondents who have experienced an injury in the previous 12 months by key characteristics such as injury type, injury severity, age, gender, and ethnic background to provide consent for interview. Where language is a barrier, an interpreter will be present. Those who participate will be provided with contact numbers for support groups should they experience any distress during their participation and would like additional support. We will offer all participants in patient interviews and focus groups a £20 voucher in recognition of their contributions [42]. We will conduct all focus groups at the premises of a local third sector organisation.

We will also conduct four focus groups with stakeholders, one in each site with up to six participants, supplemented with telephone interviews as necessary. We will recruit stakeholders from a range of professional groups: ambulance call takers, paramedics, and operational managers; ED clinicians; GPs and other primary care staff; social services staff; and third sector support workers. We will explore participants' experiences and practicalities of delivering care to patients from ethnic minorities who present with injury, including their resource and training needs. We will offer all participants a £20 voucher to acknowledge the time taken to contribute to the study [51].

WP 5: Synthesis of quantitative and qualitative findings

We will synthesise findings obtained from the four previous WPs to ensure that findings assist policymakers, commissioners and care providers to achieve the best outcomes for patients, staff and the wider health care system.

Analysis

WP1: Scoping review

We will chart data reporting on aims, sample size, demographics, injury presentation, cause of injury, definition of mortality and the difference in rate of mortality. We will describe but

For peer review only - http://bmjopen.bmj.com/site/about/guidelines.xhtml

not appraise included papers for methodological quality or risk of bias, which is consistent with guidance on conducting scoping reviews [42].

WP2: Epidemiology of injury presentation, care delivery and outcomes using anonymised routine linked data

Our Statistical Analysis Plan (SAP) will characterise and allow for differences in population between study sites. We will interpret results in the light of these differences to maximise generalisability across the UK population. We will detail conventions on comparison of processes and outcomes (including inclusion and exclusion rules for covariates and factors), management of missing data, selection of confounders and the reporting of outcomes. To ensure we can report on outcomes by ethnicity (and ethnic subgroups, where appropriate), we will cross-reference and validate key variables across data sources (e.g., Hospital Episode Statistics (HES) or ONS and CCG (England). We will adjust our comparisons between cohorts (people from ethnic minorities and white British) and subgroups using pre-specified factors and covariates (e.g., age; gender; socio-economic status) obtainable from routine data. We will request deprivation measures associated with patient residence. These socio-ecological data will comprise an Index of Multiple Deprivation and component domains and we will include appropriate summaries as confounders in our statistical models.

We will describe and compare when analysing our routine data, summarising the epidemiology of injury by ethnicity (including patterns of presentation; injury type, severity, and case-mix; processes and outcomes of care) based on those presenting to the emergency services within and between people from ethnic minorities and white British people. We will include analysis by ethnic subgroups where numbers allow. Across the four study sites, we expect to identify approximately 70,000 people from ethnic minorities and 480,000 white British people who have sought emergency care for injury. This will give ample power to

For peer review only - http://bmjopen.bmj.com/site/about/guidelines.xhtml

undertake meaningful comparisons across aspects of presentation (e.g., proportion presenting with a specific condition), disposition (e.g., proportion admitted to hospital; length of stay) and further outcomes (e.g., re-attendance rates, mortality) over time and between cohorts and pre-specified subgroups.

Limitations in routine data will define a third study cohort, comprised of people presenting with injury but for whom no useable data on ethnicity are available. We will describe the characteristics and outcomes (e.g., age, sex, injury type and severity; and health outcomes) for this cohort and compare them with the people from ethnic minorities and white British cohorts. This will address our objective related to the quality of ethnicity data in emergency care settings.

WP3: Questionnaire survey

We will collate questionnaire data on a secure platform; initial processing will include data validation, assessment of its quality and completeness, and implementation of published scoring algorithms. In our analyses we will report descriptive summaries of responses (using standard methods, including tabulated counts and percentages); comparative analyses, combining questionnaire outcomes with pre-specified factors and covariates; and, where feasible, description and comparison of questionnaire respondents and non-respondents.

WP4: Qualitative interviews and focus groups

We will use framework analysis [52] to analyse qualitative interview and focus group data. We will identify themes from our study questions, the literature and initial analysis of survey data to develop our framework. We will code transcripts according to these themes and refine as analysis progresses. Experienced qualitative researchers will lead analysis of interview and focus group transcripts. Two public contributors will help to validate the analysis process, supporting key stages of coding, refining themes and provide a critical stance [53]. We will

For peer review only - http://bmjopen.bmj.com/site/about/guidelines.xhtml

use NVivo 11, computer assisted qualitative data analysis, to manage data. We will remove all identifiable data from interview and focus groups transcripts and assign a participant number for identification. Where appropriate, anonymous coded excerpts or quotes will be included in outputs.

WP5: Synthesis of quantitative and qualitative findings

We will synthesise and report on quantitative and qualitative findings by identifying meta-themes that cut across each component of the study [54]. We will interpret the results and consider similarities and differences, including recurring themes and issues that emerge from the scoping review, routine data, survey responses and people's views and experiences of injury and care received. We will use this evidence to inform our policy recommendations for improving injury care for people from ethnic minorities, including direction of future research.

ETHICS AND DISSEMINATION

We have obtained a favourable ethical approval from the Wales Research Ethics Committee (REC) (305391). We have also completed all necessary research permissions through the Health Research Authority (HRA). In addition, we obtained information governance approvals from the Confidentiality Advisory Group (CAG) to conduct data linkage and retrieval of outcomes for analysis from NHS Digital in England (via its Data Access Request Service (DARS)) and are in the process of gaining approval from eDRIS in Scotland (University of Edinburgh and NHS Lothian).

Patient And Public Involvement

We will ensure our public contributors are actively involved in all aspects of the study [55]. We have strong relationships with people from ethnic minorities who have contributed

For peer review only - http://bmjopen.bmj.com/site/about/guidelines.xhtml

experience-based expertise throughout the process of planning this proposal. We have drawn on the experiences and knowledge of two experienced public contributors to design the study who will join the Research Management Group to implement the research (TH, SS). We will recruit two additional public contributors to join the independent Study Steering Committee alongside clinical, policy, academic, methodological and subject experts. We will also regularly present progress and emerging findings of our study to two public advisory groups, the PRIME SUPER Group [56] and the SAIL Consumer Panel [57]. We will provide honoraria, briefings and other support as needed in line with best practice and report public involvement in our outputs [58].

Dissemination

We will include engagement with patient and professional groups, NHS managers, commissioners and policy makers and third sector organisations in our communication, publication and dissemination plan. We will use the plan to guide our second Stakeholder Event, which will take place once the study data collection and analysis are complete. The Stakeholder Event will be designed to be inclusive allowing patients, public contributors, third sector organisations, service providers and policy makers the space to share their views. At the event, we will discuss and refine our findings to ensure our results are credible and are widely shared with the community and service providers.

DISCUSSION

This is the first study in the UK to use routine anonymised linked data to compare outcomes and experiences for people from ethnic minorities and white British people when they present with injury to emergency health services. Our mixed-methods design builds on this innovative approach to capturing data by employing qualitative methods (WP4) to gain an in-depth understanding of a range of experiences, outcomes, and views about emergency care

For peer review only - http://bmjopen.bmj.com/site/about/guidelines.xhtml

that are not available in routine health records. Our focus groups with asylum seekers and refugees (WP4) provide a valuable insight to the ways an already vulnerable population accesses and navigates emergency health services when contacted for care following injury.

The strength of this study lies in its multifaceted approach to study design, data collection and analysis which stems from the diverse study team. Collaborating with ambulance services, EDs, community members and third sector organisations not only strengthens the implementation of the study's research activities, but also ensures that the contribution this study makes to the evidence base will be informed by those who deliver and use emergency services.

FIGURES

Figure 1. Study design and participant recruitment flowchart 7

Figure 2. WP2 – Data flow for routine linked data epidemiology of injury presentation, care delivery and outcomes...9

Figure 3. WP3 – Data flow for questionnaire survey...13

AUTHORS CONTRIBUTION

FB drafted the manuscript with editorial input from all authors – FB, BAE, SG, TA, AK, AJ, PG, RAL, AP, ITR, SS, ANS, AW, JW, HS. The research idea was conceived by AK and HS and developed by all authors. All authors read and approved the final manuscript.

COMPETING INTERESTS STATEMENT

There are no competing interests.

For peer review only - http://bmjopen.bmj.com/site/about/guidelines.xhtml

FUNDING STATEMENT

This work was supported by National Institute for Health and Care Research grant number NIHR132744.

REFERENCES

1. Aldridge E, Sethi D, Yon Y. Injuries: a call for public health action in Europe Injuries: a call for public health action in Europe. An update using the 2015 WHO global health estimates. *WHO Regional Office for Europe.* 2015. https://www.euro.who.int/__data/assets/pdf_file/0018/348102/WH08_IJ_complete-web-version.pdf?ua=1. (Accessed 9 Jun 2022).
2. NICE. Unintentional Injuries: Prevention Strategies for under 15s, in *Public Health Guideline* [PH29]. 2010. https://www.nice.org.uk/guidance/ph29. (Accessed 9 Jun 2022).
3. NHS digital. CDS Type 011 – Emergency Care Data Set (ECDS) User guide. *NHS Digital*. 2020. https://digital.nhs.uk/data-and-information/data-collections-and-datasets/data-sets/emergency-care-data-set-ecds/user-guide/data-group-injury-characteristics?key=#5-14-1-definition-group-status-and-justification-for-data-group (Accessed 9 Jun 2022).
4. Matheson J, Patterson J, Neilson L. Tackling Causes and Consequences of Health Inequalities: A Practical Guide. London: CRC Press 2020.
5. Public Health England. Public Health Outcomes Framework: Health Equity Report - Focus on ethnicity. 2017.
6. NHS. The NHS long term plan. https://www.longtermplan.nhs.uk/. (Accessed 9 Jun 2022).
7. Honeyford K, Coughlan C, Nijman RG, Expert P, Burcea G, Maconochie I, Kinderlerer A, Cooke GS, Costelloe CE. Changes in Emergency Department Activity and the First COVID-19 Lockdown: A Cross-sectional Study. *West J Emerg Med* 2021;22(3)603–607. DOI: 10.5811/westjem.2021.2.49614

For peer review only - http://bmjopen.bmj.com/site/about/guidelines.xhtml

8. Phung VH, Siriwardena N, Windle K, et al. Ethnicity and prehospital emergency care provided by ambulance services. *Race Equality Foundation* 2015 http://eprints.lincoln.ac.uk/id/eprint/17521/1/Health%20Briefing%2037.pdf. (Accessed 9 Jun 2022).

9. Khanom A, Alanazy W, Snooks H, et al. Asylum seekers' and refugees' experiences of accessing health care: a qualitative study. *BJGP Open* 2021;14;5(6):DOI:10.3399/BJGPO .2021.0059.

10. Graetz V, Rechel B, Groot W, et al. Utilization of health care services by migrants in Europe-a systematic literature review. *Br Med Bull* 2017;121(1):5-18.

11. Gunst M, Jarman K, Yarwood V, Rokadiya S, Capsaskis L, Orcutt M, Abbara A. Healthcare access for refugees in Greece: Challenges and opportunities. *Health Policy* 2019; 1;123(9):818-24 DOI:10.1016/j.healthpol.2019.06.003

12. Satinsky E, Fuhr DC, Woodward A, Sondorp E, Roberts B. Mental health care utilisation and access among refugees and asylum seekers in Europe: a systematic review. *Health Policy* 2019;1;123(9):851-63 DOI: 10.1016/j.healthpol.2019.02.007

13. Hewes H, Dai M, Mann NC, et al. Prehospital Pain Management: Disparity By Age and Race. *Prehosp Emerg Care* 2018; 22(2):189-197. DOI: 10.1080/10903127.2017. 1367444

14. Herrera-Escobar JP, Rivero R, Apoj M, et al. Long-term social dysfunction after trauma: What is the prevalence, risk factors, and associated outcomes? *Surgery* 2019;166(3):392-397.

15. Ali I, Vattigunta S, Jang JM, et al. Racial Disparities are Present in the Timing of Radiographic Assessment and Surgical Treatment of Hip Fractures. *Clin Orthop Relat Res* 2020;478(3):455-461.

For peer review only - http://bmjopen.bmj.com/site/about/guidelines.xhtml

16. LaPlant MB, Hess DJ. A review of racial/ethnic disparities in paediatric trauma care, treatment, and outcomes. *Trauma Surg Acute Care Open* 2019;86(3):540-550.

17. Hsia RY, Markowitz AJ, Lin F, et al. Ten-year trends in traumatic brain injury: a retrospective cohort study of California emergency department and hospital revisits and readmissions. *BMJ Open* 2018;8(12): e022297.

18. Haider AH, Chang Dc, Efron ER, et al. Race and insurance status as risk factors for trauma mortality. *Arch Surg* 2008;143(10):945-949

19. Athavale AM, Fu CY, Bokhari F, et al. Incidence of, Risk Factors for, and Mortality Associated With Severe Acute Kidney Injury After Gunshot Wound. *JAMA Netw open* 2019;2(12): e1917254,DOIi:10.1001/jamanetworkopen.2019.17254

20. Meixner C, Loder RT. The Demographics of Fractures and Dislocations Across the Entire United States due to Common Sports and Recreational Activities. *Sports health* 2020; 12(2): 159-169.

21. de Angelis P, Kaufman EJ, Barie PS, et al. Disparities in Timing of Trauma Consultation: A Trauma Registry Analysis of Patient and Injury Factors. *Journal Surg Res* 2019;242:357-362.

22. Roudsari B, Caetano, R, Field, C. Alcohol intoxication/dependence, ethnicity and utilisation of health care resources in a level I trauma center. *Injury* 2011;42(1):66-71.

23. Plener PL, Muns LM, Allroggen M, et al. Immigration as risk factor for non-suicidal self-injury and suicide attempts in adolescents in Germany. *Child Adolesc Psychiatry Ment Health* 2015;9(1):34.

24. Sakran JV, Nance M, Rail T, et al. Paediatric Firearm Injuries and Fatalities: Do Racial Disparities Exist? *Ann Surg* 2020;272(4):556-561.

25. Rojas-Velasquez, DA, Pluhar EL, Burns PA, et al. Nonsuicidal Self-Injury Among African American and Hispanic Adolescents and Young Adults: a Systematic Review. *Prev Sci* 2020;22(3):367-377.

For peer review only - http://bmjopen.bmj.com/site/about/guidelines.xhtml

26. Hodes D, O'Donnell NA, Pall K, et al. Epidemiological surveillance study of female genital mutilation in the UK. *Arch Dis Child* 2020;106(4)372-376.

27. Cézard G., Gruer L, Steiner M, et al. Ethnic variations in falls and road traffic injuries resulting in hospitalisation or death in Scotland: the Scottish Health and Ethnicity Linkage Study. *Public health* 2020;182:32-38.

28. Dean E, Taylor MJ, Francis H, et al. An exploration of community and culture related fire injury risks. *UKAIS* 2016, http://researchonline.ljmu.ac.uk/id/eprint/3523/

29. Loder RT, Robinson, TP. The demographics of patients presenting for sexual assault to US emergency departments. *J Forensic Leg Med* 2020;69:101887.

30. Steinbach R, Edwards P, Green J. Controlling for exposure changes the relationship between ethnicity, deprivation and injury: an observational study of child pedestrian injury rates in London. *Inj Prev* 2014;20(3):159-166. doi:10.1136/injuryprev-2012-040741

31. Sewell T, Aderin-Pocock M, Chughtai A, et al. The report of the Commission on Race and Ethnic Disparities 2021. https://www.gov.uk/government/publications/the-report-of-the-commission-on-race-and-ethnic-disparities

32. Asghar Z., Phung VH., Siriwardena, AN. Ethnicity and pre-hospital care for people with suspected cardiac pain: cross-sectional study. *J Eval Clin Pract* 2016;22(5):721-725.

33. Gill PS, Beavan J, Calvert M, et al. The unmet need for interpreting provision in UK primary care. *PLoS one* 2011;13;6(6):e20837, DOI: 10.1371/journal.pone.0020837

34. Kamal A, Hodson A, Pearce JM. A rapid systematic review of factors influencing COVID-19 vaccination uptake in minority ethnic groups in the UK. *Vaccines* 2021;1;9(10):1121. DOI: org/10.3390/vaccines9101121

35. Memon A, Taylor K, Mohebati LM, et al. Perceived barriers to accessing mental health services among black and minority ethnic (BME) communities: a qualitative study

For peer review only - http://bmjopen.bmj.com/site/about/guidelines.xhtml

in Southeast England. *BMJ open* 2016;1;6(11):e012337, DOI: 10.1136/bmjopen-2016-012337

36. Goodacre S, Thomas B, Lee E, et al. Characterisation of 22445 patients attending UK emergency departments with suspected COVID-19 infection: Observational cohort study. *PLoS One* 2020;15(11):e0240206.

37. Creswell JW, Plano Clark VL. Chapter 3: Choosing a mixed methods design. In Creswell JW, Clark VL, eds. Designing and conducting mixed methods research. London: SAGE. 2011;(2)53-106.

38. Giddens, A. The constitution of society: Outline of the theory of structuration. *University of California Press;* 1984.

39. Ray V. A Theory of Racialized Organizations. *Am Sociol Rev* 2019;84(1):26-53. DOI: 10.1177/0003122418822335

40. Peters MD, Godfrey CM, Khalil H, et al. Guidance for conducting systematic scoping reviews. *Int J Evid Based Healthc* 2015;13(3):141-6. DOI: 10.1097/XEB.0000000000 000050.

41. Tricco AC, Lillie E, Zarin W, et al. PRISMA Extension for Scoping Reviews (PRISMAScR): Checklist and Explanation. *Ann Intern Med* 2018;169:467–473. DOI: 10.7326/M18-0850.

42. Whiting P, Rutjes AWS, Reitsma JB, et al. The development of QUADAS: a tool for the quality assessment of studies of diagnostic accuracy included in systematic reviews. *BMC Med Res Methodol* 2003;3:25 DOI:10.1186/1471-2288-3-25.

43. Mourby MJ, Doide KH, Jones, et al. Health Data Linkage for UK Public Interest Research: Key Obstacles and Solutions. *Int J Popul Data Sci* 2019;4(1):1093-1093.

44. Lyons RA, Jones KH, John G, et al. The SAIL databank: linking multiple health and social care datasets. *BMC Med Inform Decis Mak* 2009;9(1):1-8.

For peer review only - http://bmjopen.bmj.com/site/about/guidelines.xhtml

45. Piel FB, Parkers BL, Daby H, et al. The challenge of opt-outs from NHS data: a small-area perspective. *J Public Health (Oxf)* 2018;40(4):e594-e600.

46. Aspinall PJ. The utility and validity for public health of ethnicity categorization in the 1991, 2001 and 2011 British Censuses. *Public Health* 2011;125(10):680-687.

47. Edwards PJ, Roberts I, Clarke MK, et al. Methods to increase response to postal and electronic questionnaires. *Cochrane database of systematic reviews* 2009(3):MR000008.

48. Carey RG, Seibert JH. A patient survey system to measure quality improvement: questionnaire reliability and validity. *Med Care* 1993:834-845.

49. Jenkinson C, Chandola T, Coulter A, et al. An assessment of the construct validity of the SF-12 summary scores across ethnic groups. *J Public Health Med* 2001;1;23(3):187-94: DOI: 10.1093/pubmed/23.3.187

50. Salway S, Chowbey P, Such E, et al. Researching health inequalities with Community Researchers: practical, methodological and ethical challenges of an 'inclusive' research approach. *Res Involv Engagem* 2015;1(9) DOI: 10.1186/s40900-015-0009-4

51. Evans BA, Bedson E, Bell P, et al. Involving service users in trials: developing a standard operating procedure. *Trials* 2013;14(1):1-7.

52. Ziebland S, McPherson A. Making sense of qualitative data analysis: an introduction with illustrations from DIPEx (personal experiences of health and illness). *Med Educ* 2006;40(5):405-414.

53. Frost J, Gibson A, Harris-Golesworthy F, et al. Patient involvement in qualitative data analysis in a trial of a patient-centred intervention: reconciling lay knowledge and scientific method. *Health Expec.* 2018;21(6):1111-21. DOI:10.1111/hex.12814

54. O'Cathain A, Murphy E, Nicholl J. Three techniques for integrating data in mixed methods studies. *BMJ* 2010;17;341. DOI: 10.1136/bmj.c4587

55. Bovaird, T. Beyond engagement and participation: User and community coproduction of public services. *Public Adm Rev* 2007;67(5):846-860.

For peer review only - http://bmjopen.bmj.com/site/about/guidelines.xhtml

56. Evans BA, Gallanders J, Griffiths L, et al. Public involvement and engagement in primary and emergency care research: the story from PRIME Centre Wales. *IJPDS*. 2020;5(3):1363. DOI: 10.23889/ijpds.v5i3.1363

57. Jones KH, McNerney CL, Ford DV. Involving consumers in the work of a data linkage research unit. *Int J Consum Stud* 2013;38(1)45-51DOI:10.1111/ijcs.12062

58. Staniszewska S, Brett J, Simera I, et al. GRIPP2 reporting checklists: tools to improve reporting of patient and public involvement in research BMJ 2017; 358 DOI: 10.1136/bmj.j3453

For peer review only

For peer review only - http://bmjopen.bmj.com/site/about/guidelines.xhtml

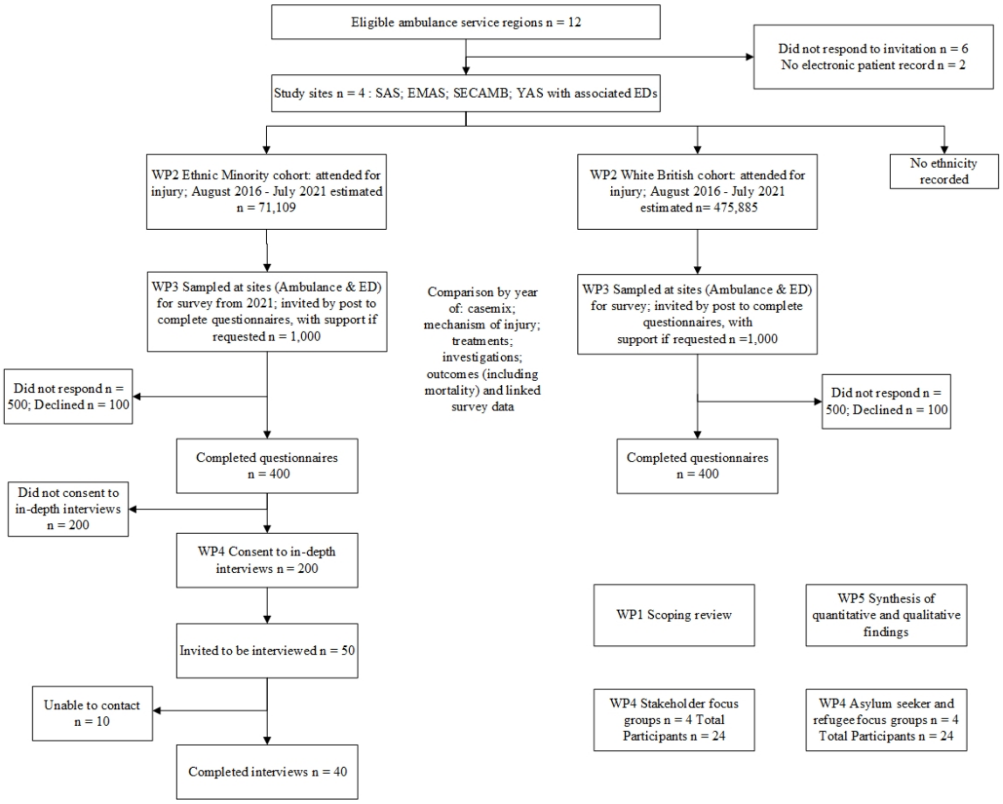

Figure 1. Study design and participant recruitment flowchart

309x246mm (96 x 96 DPI)

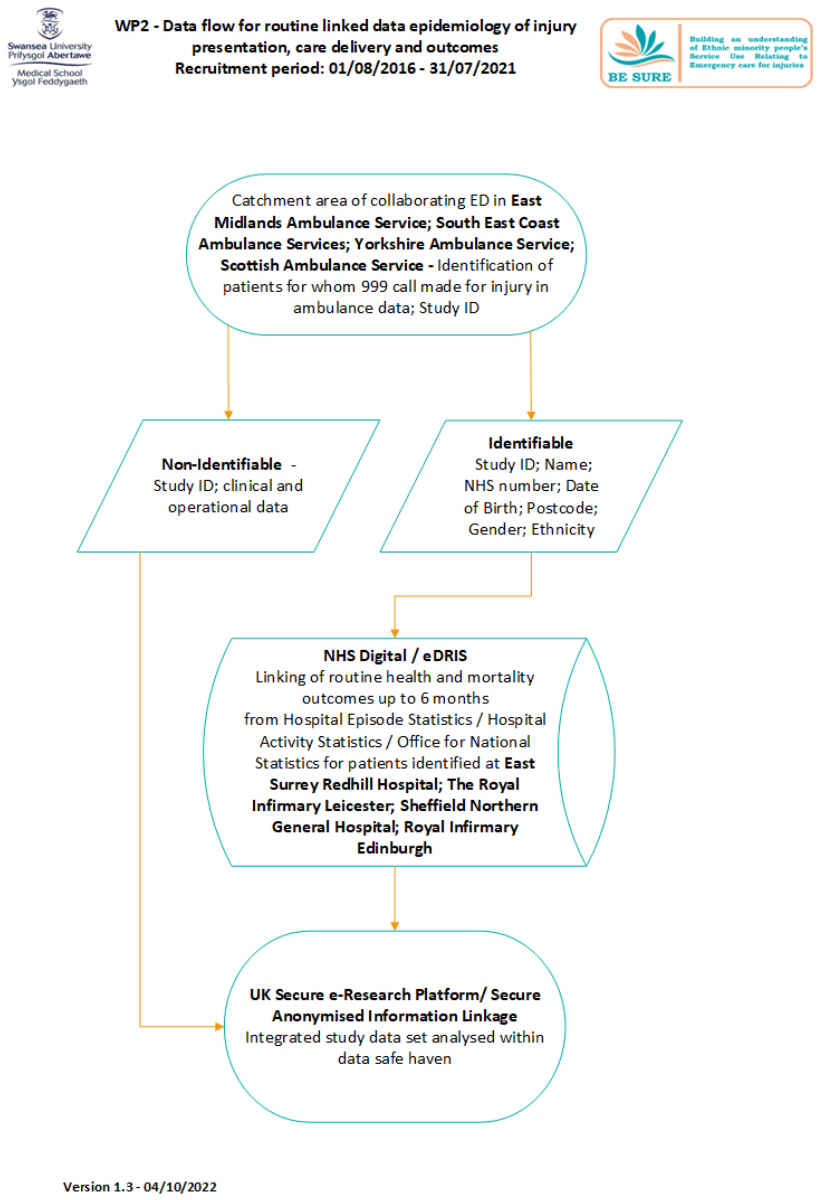

Figure 2. WP2 – Data flow for routine linked data epidemiology of injury presentation, care delivery and outcomes

193x283mm (96 x 96 DPI)

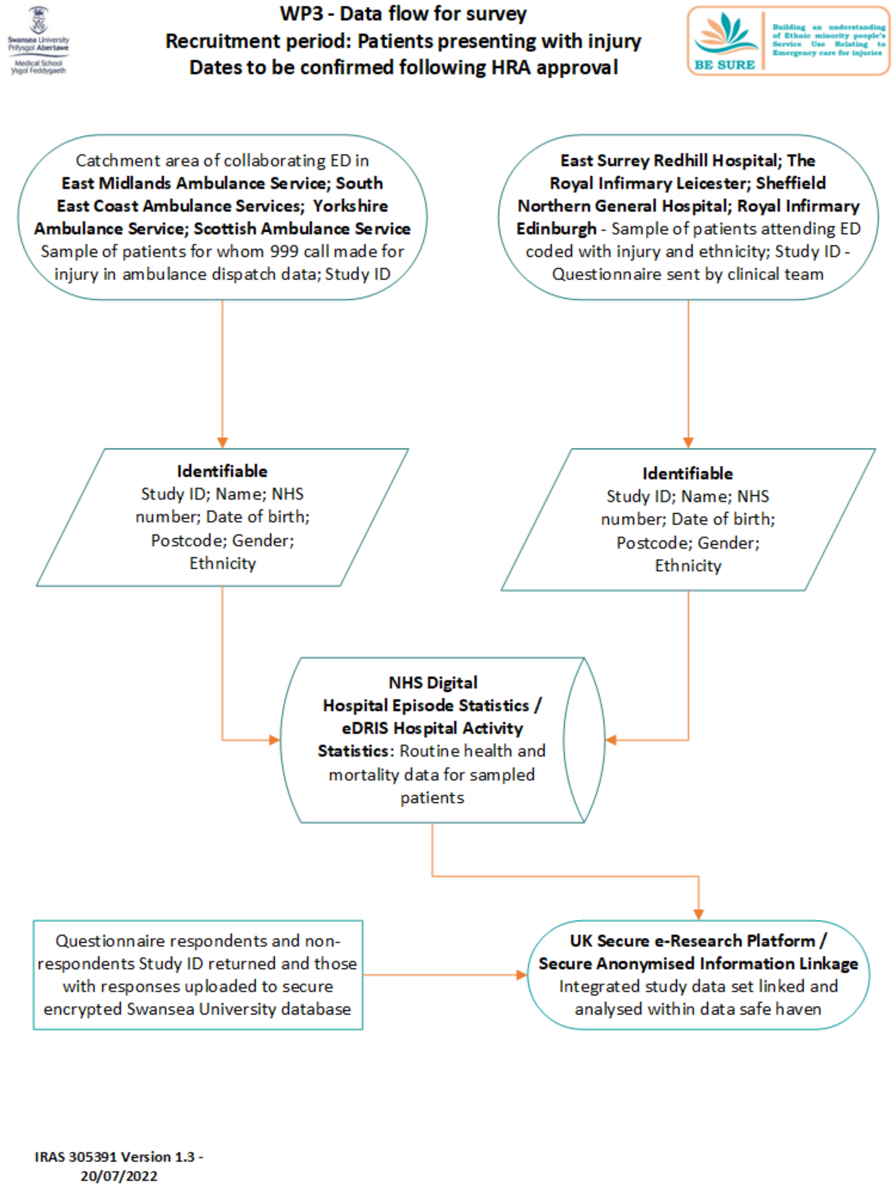

Figure 3. WP3 – Data flow for questionnaire survey

197x265mm (96 x 96 DPI)

TREND Statement Checklist

Paper Section/ Topic	Item No	Descriptor	Reported? ✓	Pg #
**Title and Abstract**				
Title and Abstract	1	• Information on how unit were allocated to interventions	✓	1
		• Structured abstract recommended	✓	1
		• Information on target population or study sample	✓	1
**Introduction**				
Background	2	• Scientific background and explanation of rationale	✓	3-4
		• Theories used in designing behavioral interventions	✓	6
**Methods**				
Participants	3	• Eligibility criteria for participants, including criteria at different levels in recruitment/sampling plan (e.g., cities, clinics, subjects)	✓	7
		Method of recruitment (e.g., referral, self-selection), including the sampling method if a systematic sampling plan was implemented	✓	8-14
		Recruitment setting	✓	5-6
		Settings and locations where the data were collected	✓	5-6
Interventions	4	• Details of the interventions intended for each study condition and how and when they were actually administered, specifically including:		
		○ Content: what was given?		
		○ Delivery method: how was the content given?		
		○ Unit of delivery: how were the subjects grouped during delivery?		
		○ Deliverer: who delivered the intervention?		
		○ Setting: where was the intervention delivered?		
		○ Exposure quantity and duration: how many sessions or episodes or events were intended to be delivered? How long were they intended to last?		
		○ Time span: how long was it intended to take to deliver the intervention to each unit?		
		○ Activities to increase compliance or adherence (e.g., incentives)		
Objectives	5	• Specific objectives and hypotheses	✓	4-5
Outcomes	6	• Clearly defined primary and secondary outcome measures	✓	5
		• Methods used to collect data and any methods used to enhance the quality of measurements	✓	6-7
		• Information on validated instruments such as psychometric and biometric properties		
Sample Size	7	How sample size was determined and, when applicable, explanation of any interim analyses and stopping rules	✓	5
Assignment Method	8	Unit of assignment (the unit being assigned to study condition, e.g., individual, group, community)		
		Method used to assign units to study conditions, including details of any restriction (e.g., blocking, stratification, minimization)		
		Inclusion of aspects employed to help minimize potential bias induced due to non-randomization (e.g., matching)		
Blinding (masking)	9	• Whether or not participants, those administering the interventions, and those assessing the outcomes were blinded to study condition assignment; if so, statement regarding how the blinding was accomplished and how it was assessed.		

For peer review only - http://bmjopen.bmj.com/site/about/guidelines.xhtml

TREND Statement Checklist

Unit of Analysis	10	• Description of the smallest unit that is being analyzed to assess intervention effects (e.g., individual, group, or community)		
		• If the unit of analysis differs from the unit of assignment, the analytical method used to account for this (e.g., adjusting the standard error estimates by the design effect or using multilevel analysis)		
Statistical Methods	11	• Statistical methods used to compare study groups for primary methods outcome(s), including complex methods of correlated data	✓	15-16
		• Statistical methods used for additional analyses, such as a subgroup analyses and adjusted analysis	✓	15-16
		Methods for imputing missing data, if used	✓	15-16
		Statistical software or programs used	✓	15-16
**Results**				
Participant flow	12	Flow of participants through each stage of the study: enrollment, assignment, allocation, and intervention exposure, follow-up, analysis (a diagram is strongly recommended)	✓	7, 8-13
		○ Enrollment: the numbers of participants screened for eligibility, found to be eligible or not eligible, declined to be enrolled, and enrolled in the study	✓	7, 8-13
		○ Assignment: the numbers of participants assigned to a study condition	✓	7, 8-13
		○ Allocation and intervention exposure: the number of participants assigned to each study condition and the number of participants who received each intervention	✓	7, 8-13
		○ Follow-up: the number of participants who completed the follow-up or did not complete the follow-up (i.e., lost to follow-up), by study condition	✓	7, 8-13
		○ Analysis: the number of participants included in or excluded from the main analysis, by study condition	✓	7, 8-13
		• Description of protocol deviations from study as planned, along with reasons		
Recruitment	13	Dates defining the periods of recruitment and follow-up	✓	6-7, 8-13
Baseline Data	14	Baseline demographic and clinical characteristics of participants in each study condition	✓	7, 8-14
		Baseline characteristics for each study condition relevant to specific disease prevention research		
		Baseline comparisons of those lost to follow-up and those retained, overall and by study condition	✓	8-14
		Comparison between study population at baseline and target population of interest	✓	8-13
Baseline equivalence	15	• Data on study group equivalence at baseline and statistical methods used to control for baseline differences	✓	8-13
Numbers analyzed	16	• Number of participants (denominator) included in each analysis for each study condition, particularly when the denominators change for different outcomes; statement of the results in absolute numbers when feasible	✓	8-13, 15-16
		• Indication of whether the analysis strategy was "intention to treat" or, if not, description of how non-compliers were treated in the analyses		

For peer review only

TREND Statement Checklist

Outcomes and estimation	17	• For each primary and secondary outcome, a summary of results for each estimation study condition, and the estimated effect size and a confidence interval to indicate the precision	✓	15-16
		• Inclusion of null and negative findings	✓	15-16
		Inclusion of results from testing pre-specified causal pathways through which the intervention was intended to operate, if any		
Ancillary analyses	18	• Summary of other analyses performed, including subgroup or restricted analyses, indicating which are pre-specified or exploratory	✓	15-16
Adverse events	19	• Summary of all important adverse events or unintended effects in each study condition (including summary measures, effect size estimates, and confidence intervals)		
**DISCUSSION**				
Interpretation	20	• Interpretation of the results, taking into account study hypotheses, sources of potential bias, imprecision of measures, multiplicative analyses, and other limitations or weaknesses of the study	✓	18-19
		Discussion of results taking into account the mechanism by which the intervention was intended to work (causal pathways) or alternative mechanisms or explanations		
		Discussion of the success of and barriers to implementing the intervention, fidelity of implementation	✓	18-19
		Discussion of research, programmatic, or policy implications	✓	18-19
Generalizability	21	• Generalizability (external validity) of the trial findings, taking into account the study population, the characteristics of the intervention, length of follow-up, incentives, compliance rates, specific sites/settings involved in the study, and other contextual issues		
Overall Evidence	22	• General interpretation of the results in the context of current evidence and current theory	✓	3-4

From: Des Jarlais, D. C., Lyles, C., Crepaz, N., & the Trend Group (2004). Improving the reporting quality of nonrandomized evaluations of behavioral and public health interventions: The TREND statement. *American Journal of Public Health*, 94, 361-366. For more information, visit: http://www.cdc.gov/trendstatement/

For peer review only

BMJ Open

Building an understanding of Ethnic minority people's Service Use Relating to Emergency care for injuries: the BE SURE study protocol

Journal:	*BMJ Open*
Manuscript ID	bmjopen-2022-069596.R1
Article Type:	Protocol
Date Submitted by the Author:	14-Mar-2023
Complete List of Authors:	Baghdadi, Fadi; Swansea University, Medical School<br>Bell, Fiona; Yorkshire Ambulance Service NHS Trust<br>Evans, Bridie; Swansea University, Swansea University Medical School Medicine<br>Goodacre, Steve; University of Sheffield, Medical Care Research Unit<br>Hettiarachchi, Thanuja; Swansea University, Public contributor, c/o Medical School<br>John, Ann; Swansea University, Medical sChool<br>John, Paul; Scottish Ambulance Service<br>Khanom, Ashrafunnesa; Swansea University, School of Medicine<br>Lyons, Ronan; University of Wales Swansea, Swansea Clinical School<br>Siriwardena, Aloysius; University of Lincoln, Community and Health Research Unit<br>Porter, Alison; University of Swansea, College of Medicine<br>Russell, Ian; Swansea University, Medicine<br>Safari, Solmaz; Swansea University, Public contributor, c/o Medical School<br>Watkins, Alan; Swansea University, College of Medicine<br>Williams, Julia; University of Hertfordshire<br>Snooks, Helen; Swansea University, Medicine
**Primary Subject Heading**:	Emergency medicine
Secondary Subject Heading:	Health services research, Public health, Qualitative research
Keywords:	ACCIDENT & EMERGENCY MEDICINE, PUBLIC HEALTH, QUALITATIVE RESEARCH

SCHOLARONE™ Manuscripts

Title Page

Building an understanding of Ethnic minority people's Service Use Relating to Emergency care for injuries: the BE SURE study protocol

Fadi Baghdadi, Medical School, Swansea University, Swansea, United Kingdom

Fiona Bell, Yorkshire Ambulance Service NHS Trust, Sheffield, United Kingdom

Bridie Angela Evans, Medical School, Swansea University, Swansea, United Kingdom

Steve Goodacre, School of Health and Related Research, University of Sheffield, Sheffield, United Kingdom

Thanuja Hettiarachchi, Public contributor, c/o Medical School, Swansea University, Swansea, United Kingdom

Ann John, Medical School, Swansea University, Swansea, United Kingdom

John Paul Anthony, Research and Innovation Hub, Scottish Ambulance Service, Edinburgh, United Kingdom

Ashrafunnesa Khanom, Medical School, Swansea University, Swansea, United Kingdom

Ronan Lyons, Medical School, Swansea University, Swansea, United Kingdom

Aloysius Niroshan Siriwardena AN, School of Health & Social Care, University of Lincoln, Lincoln, United Kingdom

Alison Porter, Medical School, Swansea University, Swansea, United Kingdom

Ian Russell, Medical School, Swansea University, Swansea, United Kingdom

Solmaz Safari, Public contributor, c/o Medical School, Swansea University, Swansea, United Kingdom

Alan Watkins, Medical School, Swansea University, Swansea, United Kingdom

Julia Williams, University of Hertfordshire, Hertfordshire, United Kingdom

Helen Snooks, Medical School, Swansea University, Swansea, United Kingdom

Corresponding author: Fadi Baghdadi

Address: Office – 205, Second Floor, Institute of Life Science 2, Singleton Campus, Sketty, Swansea, SA2 8PP, United Kingdom.

Email: fadi.baghdadi@swansea.ac.uk

Phone number: +44 1792 513409

Word Count = 3,896

For peer review only - http://bmjopen.bmj.com/site/about/guidelines.xhtml

Building an understanding of Ethnic minority people's Service Use Relating to Emergency care for injuries: the BE SURE study protocol

ABSTRACT

Introduction

Injuries are a major public health problem which can lead to disability or death. However, little is known about the incidence, presentation, management and outcomes of emergency care for patients with injuries among people from ethnic minorities in the United Kingdom. The aim of this study is to investigate what may differ for people from ethnic minorities compared to white British people when presenting with injury to ambulance and emergency departments (EDs).

Methods and Analysis

This mixed methods study covers eight services, four ambulance services (three in England and one in Scotland) and four hospital EDs, located within each ambulance service. The study has five work packages (WP): (WP1) scoping review comparing mortality by ethnicity of people presenting with injury to emergency services; (WP2) retrospective analysis of linked NHS routine data from patients who present to ambulances or EDs with injury over five years (2016-2021); (WP3) postal questionnaire survey of 2,000 patients (1,000 patients from ethnic minorities and 1,000 white British patients) who present with injury to ambulances or EDs including self-reported outcomes (measured by Quality of Care Monitor and Health Related Quality of Life measured by SF-12); (WP4) qualitative interviews with patients from ethnic minorities (n = 40) and focus groups – four with asylum seekers and refugees and four with care providers; and (WP5) a synthesis of quantitative and qualitative findings.

Ethics and Dissemination

This study received a favourable opinion by the Wales Research Ethics Committee (305391). The Health Research Authority has approved the study and, on advice from the Confidentiality Advisory Group, has supported the use of confidential patient information without consent for anonymised data. Results will be shared with Ambulance and ED services, government bodies and third sectors organisations through direct communications summarising scientific conference proceedings and publications.

For peer review only - http://bmjopen.bmj.com/site/about/guidelines.xhtml

Strengths and limitations of this study

Strengths:

- Mixed quantitative and qualitative methods will ensure representativeness and depth in addressing our research questions
- The study will use peer researchers from ethnic minorities recruited by local third sector organisations to support people to complete questionnaires to ensure local buy in and to achieve as high a response rate to the survey as possible
- Routine linked data allows the inclusion of a large number of patients and attendances over a 5-year period, producing a comprehensible epidemiological picture

Limitations:

- Coding of ethnicity may be inaccurately recorded or incomplete in routine health records which may mask heterogeneity within each group
- Response rates to questionnaire surveys may be low and differ between cohorts, introducing potential bias to findings

Keywords

Emergency Medical Services

Wounds and Injuries

Electronic Health Records

Medical Record Linkage

Minority Health

Ethnicity in Health Services

For peer review only

INTRODUCTION

Injuries cause 5 million deaths worldwide each year and many more people are left with disability [1]. In the United Kingdom (UK) around six million Emergency Department (ED) visits result from accidental injuries each year [2] and over 14,000 of these injuries result in death [3].

A founding and sustained principle of the National Health Service (NHS) is that there should be equity of access and treatment for all [4]. However, disparities in access to health care and health outcomes for people from ethnic minorities compared to white British people have been regularly reported[5]. Future changes in the delivery of NHS care as proposed in the NHS Long Term Plan [6] may deepen inequalities, as people with urgent care needs including minor injuries are redirected towards NHS 111 (telephone service) and GP led Urgent Treatment Centres. Following COVID-19, further initiatives have been trialled to control immediate access to emergency care[7]. However, there are concerns that people from ethnic minorities are more likely to make greater use of emergency healthcare, reflecting difficulties in accessing primary care [8]. In the Health Experiences of Asylum Seekers and Refugees (HEAR) study, [9] 77% of survey respondents knew about the 999 service, but only 28% were aware of the Out of Hours General Practitioner (GP) service. Research across Europe [10] reports a rise in migrants' and asylum seekers' use of emergency services. High use has been associated with language barriers, social deprivation, poor access to primary care [10], delayed or restricted access to secondary healthcare [11], or people falling through gaps between other services (such as community mental health services) [12].

People from ethnic minorities across Europe, North America and Oceania have been widely reported to have differences in access, experiences, and outcomes when presenting to

For peer review only - http://bmjopen.bmj.com/site/about/guidelines.xhtml

emergency services. People from ethnic minorities who present with injuries have different experiences in relation to pain management [13], length of hospital stay [14], quality of care [15], disability [16], repeat attendance [17], and mortality [18]. They also have increased risk of certain injury presentations including; gunshot injuries [19], long bone fractures [20], head injuries [21], alcohol related injury [22], workplace injury [23], assaults [24], self-harm and attempted suicide [25] and Female Genital Mutilation (FGM) [26]. However, people from ethnic minorities have lower prevalence of other injuries including: falls among the elderly and road traffic injuries [27], fire injuries [28] and partner violence [29].

While death and morbidity rates due to injury are higher in some ethnic minority populations in the UK [30], there remains a gap in evidence on their experiences of emergency services. This is partly due to a lack of focus or priority on this area of inequality until recently [31]. Firstly, there is a weakness in routine information systems, where ethnicity data are often poorly recorded, particularly in emergency prehospital care settings [32]. Secondly, the preferred language of patients from ethnic minorities are not recorded in routine health data, nor are differences in culture and language adequately accommodated for in emergency services, with a scarcity of government funded interpreters [33], public health campaigns [34] and allied health services [35]. There is considerable scope for taking a more analytical approach to studying injury presentation and differences in emergency care among people from ethnic minorities in the UK that will inform policy and practice and help to reduce future disparities and burden of injury, mortality and disability.

Study Aim

To describe disparities in injury presentation, processes of care and outcomes between people from ethnic minorities and white British people when they contact emergency health services for injury.

For peer review only - http://bmjopen.bmj.com/site/about/guidelines.xhtml

Objectives

We will:

1) Describe the published literature reporting all-cause mortality of people presenting with injury to emergency services by ethnicity
2) Describe the quality (completeness, consistency) of ethnicity data in routine emergency healthcare datasets
3) Compare between people from ethnic minorities and white British people: injury type, severity, care delivered, outcomes, beliefs and experiences when they contact emergency health services for injuries
4) Explore with people from ethnic minorities, including refugees and asylum seekers: knowledge of service availability, factors which deter or encourage them to seek help, experiences of emergency healthcare for injuries
5) Explore emergency healthcare providers' experiences of delivering care to people from ethnic minorities presenting with injury
6) Synthesise quantitative and qualitative findings to:
    a. Help policy makers and care providers to develop and implement interventions to promote accessibility of services for injury in ethnic minorities populations
    b. Enable ambulance service and EDs to improve care and outcomes for people in these populations with injuries
    c. Inform injury surveillance resources to include ethnicity in their reporting of injury

METHODS

Setting

We will conduct this study in the catchment area of one receiving hospital ED within each of four ambulance services (Table 1.). We selected sites where an established electronic patient data capture system was in place in the ambulance service. The participating ambulance services will provide linkable electronic datasets including ethnicity codes, which are available in approximately 70% of patient records [32]. We hope to retrieve 80% of centrally

For peer review only - http://bmjopen.bmj.com/site/about/guidelines.xhtml

For peer review only

held ED records with ethnicity codes [36]. The study will begin on the 01/10/2021 and end on the 30/09/2023.

Ambulance service	Emergency Department	Third Sector Organisation
East Midlands Ambulance Services	Leicester Royal Infirmary, NHS Trust	The Race Equality Centre
South East Coast Ambulance service	East Surrey Hospital, Surrey and Sussex Healthcare, NHS Trust	Surrey Minority Ethnic Forum
Scottish Ambulance Service	Royal Infirmary of Edinburgh, History, NHS Lothian	The Welcoming
Yorkshire Ambulance Service	Northern General Hospital, Sheffield Teaching Hospitals, NHS Foundation Trust	Refugee Council

Table 1. Study sites and partners

Third sector organisations who provide support in relation to health care for people from ethnic minorities at each site will help connect researchers with the local population. They will promote the study across their networks to encourage people to respond to postal survey questionnaires and provide support with recruiting and managing peer researchers to support sampled patients to complete the questionnaires.

Study Design

We will use a convergent model of data collection where the quantitative and qualitative methods are conducted in parallel, and results are merged during the interpretation stage [37]. We will examine disparities in experiences, injury presentation, processes of care and outcomes as both the product of the individual patients' decisions and actions [38], and the organisations decisions, actions and attitudes [39]. As shown in Figure 1, in our Work Packages (WP) we will:

WP1) Conduct a scoping review of existing literature.

For peer review only - http://bmjopen.bmj.com/site/about/guidelines.xhtml

WP2) Retrieve and analyse retrospective linked NHS routine data over a five-year period (2016-2021) related to ambulance and ED contacts by patients from ethnic minorities and white British patients for injury to compare demographics, casemix, processes and outcomes of care.

WP3) Conduct a questionnaire survey with samples of people from ethnic minorities and white British people who contacted the ambulance service or attended ED for an injury within a specified recent period of up to 6 months to compare self-reported experiences, satisfaction and health related quality of life.

WP4) Conduct in-depth interviews in each site with people from ethnic minorities who consent to be contacted for an interview in their completed questionnaires and conduct one focus group with refugees and/or asylum seekers at each site and one focus group with stakeholders at each site: e.g., ED clinicians, paramedics, GPs and other primary care staff, social services staff, and third sector support workers.

WP5) Synthesise our findings from quantitative and qualitative data to generate key messages and implications for policy and service delivery.

Figure 1. Study design and participant recruitment flowchart

WP 1: Scoping review

We will undertake a scoping review following the Joanna Briggs Institute (JBI) methodology [40]. We will refer to the Preferred Reporting Items for Systematic Reviews and Meta-analysis Extension for Scoping Reviews (PRISMA - ScR) [41] and JBI reporting checklists to developing a scoping review protocol [42]. The scoping review will aim to describe the published literature reporting cases of mortality by race or ethnicity of adults presenting with injury to emergency services.

For peer review only - http://bmjopen.bmj.com/site/about/guidelines.xhtml

We will identify papers from database searches (EBSCO = CINAHL, MEDLINE and PsycInfo; SCOPUS and COCHRANE) which will be screened independently by title, abstract and full paper following a protocol by at least two reviewers from the research team (See supplementary file 1). We will include studies that report all cases of mortality by race or ethnicity of adults presenting to emergency services for injury. We will exclude studies involving people with no ethnicity record; no record of injury as the cause of emergency service use; no reporting of mortality by race and ethnicity and those reporting non-emergency care such as scheduled appointments, outpatient department services and elective surgeries. All discrepancies between reviewers will be resolved by a third reviewer.

WP 2: Epidemiology of injury presentation, care delivery and outcomes using anonymised linked routine data

We will link routine ambulance service data between August 2016 and July 2021 related to patients presenting with injury within the ambulance service catchment area of each participating ED to centrally held ED, inpatient, outpatient and Office of National Statistics (ONS) datasets (using NHS Digital in England and eDRIs in Scotland) [43]. Individual-level prehospital data on calls made for injury will be retrieved by each ambulance service from its Computer Aided Dispatch (CAD) and Patient Clinical Record (PCR) systems; these data are currently unavailable in NHS Digital or Electronic Data Research and Innovation Service (eDRIS). Clinical data will include ethnicity; condition code; job cycle time (from first 999 call for the incident to time ambulance reported free to respond to next 999 call); medications given and disposition (conveyed to hospital, treated without conveyance). We will link this data using a study-specific Identity (ID) to patient identifiable data held in separate files – the "split file" method [44] – and uploaded to NHS Digital or eDRIS by each site. We will then use patient identifiable data within NHS Digital or eDRIS to create Anonymised Linkage Fields (ALFs) and retrieve routinely recorded outcomes for these patients (Figures 2).

For peer review only - http://bmjopen.bmj.com/site/about/guidelines.xhtml

Figure 2. WP2 – Data flow for routine linked data epidemiology of injury presentation, care delivery and outcomes

We will also retrieve routine data on ED attendances for injury from participating EDs for the same period from NHS Digital / eDRIS. We will then retrieve anonymised linked routine health outcomes for six months after index presentation with injury to ambulance services and EDs, to assess outcomes unless the person has specifically opted out [45]. We will request data related to: diagnoses; disposition from ambulance service and ED; length of stay at index episode in hospital; treatments received and discharge code; Intensive Care Unit (ICU) admissions and length of stay; further ED attendances and emergency admissions; and deaths up to six months.

We will partition the aggregated data into cohorts of patients from ethnic minorities, patients from the white British population and those for whom no ethnicity is recorded; with appropriate sub-groups identified using 2011 Census ethnicity categories [46]. We will include patients with multiple presentations or attendances with the first presentation or attendance as baseline, and data from subsequent presentations or attendances contributing to outcomes. We will hold a consultation workshop with stakeholders at the outset of the study to help clarify and define our study outcome measures.

We will compare patterns of presentation, processes of care and outcomes through cross-sectional analyses to investigate differences in:

- Demographics; geography and deprivation index; mechanism of injury; severity; injury type (accidental, non-accidental, assault, self-harm); casemix; route to care (direct, via 111 telephone advice service or via general practice)
- Treatments and investigations

For peer review only - http://bmjopen.bmj.com/site/about/guidelines.xhtml

- Potential safety incidents (e.g., hospital admission or death within 72 hours of discharge from 999 or ED care) following injury
- Immediate outcomes (at index event) including ambulance attendance, transportation to hospital, hospital admission, length of stay in hospital and ICU, death following injury
- 6-month outcomes (further ED or hospital attendances, length of stay in hospital and deaths) following injury

WP 3: Questionnaire survey

We will survey people from ethnic minorities and white British people who have presented with injury to one of the four ambulance services or nominated ED. Each of the four study sites will search through their routine ambulance service and ED records to identify patients presenting with injury and coded as being from an ethnic minority, including patients who presented to the ambulance service but were not conveyed to hospital. They will also identify a similar sized cohort of patients identified as white British.

Each study site (comprising one ambulance service and one ED) will send out 500 postal questionnaires (n=2000 in total, 4 sites) to 250 patients from ethnic minorities and 250 white British patients. Before sending out questionnaires, the clinical care team will check death records to ensure that the person has not died, to avoid causing distress to their family. All recipients will be asked to return completed questionnaires in a prepaid envelope to Swansea University. A reminder letter will be sent after two weeks. Recipients will also have the option to complete the questionnaire online (via a secure approved platform) using a QR code, reducing the potential burden of having to return the questionnaire by post. Where patient contact numbers are available, the clinical care team will contact the patient by telephone one week after sending the questionnaire, to offer support to complete the questionnaire over the telephone, or to refer the participant to a local peer researcher to

For peer review only

For peer review only - http://bmjopen.bmj.com/site/about/guidelines.xhtml

provide this support where consent is provided. The expected 800 analysable questionnaire responses will (using 90% power, 5% significance) enable us to detect differences in outcomes equivalent to a standardised statistical effect of ~0.23; this, in turn, corresponds to clinically meaningful differences in study outcomes (e.g., self-reported Health Related Quality of Life). We will offer all respondents a £10 voucher for completing the questionnaire [47]. All questionnaire data will be inputted and stored on secure Swansea University database.

We will base our survey questions on those used successfully in the HEAR survey,**Error! Bookmark not defined.** focusing on knowledge of services, beliefs, experiences of injury, expectations, and health-seeking behaviour. The questionnaire (See supplementary file 2) will also include standardised questionnaires to measure satisfaction with care (Quality of Care Monitor) [48] and current health status (SF-12) [49]. The questionnaire will be translated into several languages and translated versions will be available on request.

We will recruit and train twelve community peer researchers [50] from ethnic minorities to support with the collection of questionnaire data. We will recruit and train peer researchers with support from our third sector partner organisations at each site. The peer researchers will work closely with the clinical care team in their localities who will refer respondents who request language support to the appropriate peer researcher with the patient's consent to help complete the questionnaire.

Identifiable data for all patients who are sampled to receive the questionnaire will be saved by participating services in a "split file" format and uploaded into NHS Digital or eDRIS (Figure 3). Questionnaire responses will be linked to clinical records from ED (HES and Emergency Care Data Set (ECDS) (England); Hospital Activity Statistics (HAS) and

For peer review only - http://bmjopen.bmj.com/site/about/guidelines.xhtml

Ambulance & Emergency Datamart (A&ED) (Scotland) and hence to factors and covariates derived from these data sources.

Figure 3. WP3 – Data flow for questionnaire survey

WP 4: Qualitative interviews and focus groups

We will conduct a total of 40 interviews with patients from ethnic minorities (10 in each site, identified from survey respondents who provide their consent and contact details) and four focus groups with Asylum Seekers and Refugees (up to six participants per site, identified by third sector organisations).

We will purposively sample survey respondents who have experienced an injury in the previous 12 months by key characteristics such as injury type, injury severity, age, gender, and ethnic background to provide consent for interview (See supplementary file 3). Where language is a barrier, an interpreter will be present. Those who participate will be provided with contact numbers for support groups should they experience any distress during their participation and would like additional support. We will offer all participants in patient interviews and focus groups a £20 voucher in recognition of their contributions [42]. We will conduct all focus groups at the premises of a local third sector organisation.

We will also conduct four focus groups with stakeholders, one in each site with up to six participants, supplemented with telephone interviews as necessary. We will recruit stakeholders from a range of professional groups: ambulance call takers, paramedics, and operational managers; ED clinicians; GPs and other primary care staff; social services staff; and third sector support workers. We will explore participants' experiences and practicalities of delivering care to patients from ethnic minorities who present with injury, including their

For peer review only - http://bmjopen.bmj.com/site/about/guidelines.xhtml

resource and training needs. We will offer all participants a £20 voucher to acknowledge the time taken to contribute to the study [51].

WP 5: Synthesis of quantitative and qualitative findings

We will synthesise findings obtained from the four previous WPs to ensure that findings assist policymakers, commissioners and care providers to achieve the best outcomes for patients, staff and the wider health care system.

Analysis

WP1: Scoping review

We will chart data reporting on aims, sample size, demographics, injury presentation, cause of injury, definition of mortality and the difference in rate of mortality. We will describe but not appraise included papers for methodological quality or risk of bias, which is consistent with guidance on conducting scoping reviews [42].

WP2: Epidemiology of injury presentation, care delivery and outcomes using anonymised routine linked data

Our Statistical Analysis Plan (SAP) will characterise and allow for differences in population between study sites. We will interpret results in the light of these differences to maximise generalisability across the UK population. We will detail conventions on comparison of processes and outcomes (including inclusion and exclusion rules for covariates and factors), management of missing data, selection of confounders and the reporting of outcomes. To ensure we can report on outcomes by ethnicity (and ethnic subgroups, where appropriate), we will cross-reference and validate key variables across data sources (e.g., Hospital Episode Statistics (HES) or ONS and CCG (England). We will adjust our comparisons between cohorts (people from ethnic minorities and white British) and subgroups using pre-specified

For peer review only - http://bmjopen.bmj.com/site/about/guidelines.xhtml

factors and covariates (e.g., age; gender; socio-economic status) obtainable from routine data. We will request deprivation measures associated with patient residence. These socio-ecological data will comprise an Index of Multiple Deprivation and component domains and we will include appropriate summaries as confounders in our statistical models.

We will describe and compare when analysing our routine data, summarising the epidemiology of injury by ethnicity (including patterns of presentation; injury type, severity, and case-mix; processes and outcomes of care) based on those presenting to the emergency services within and between people from ethnic minorities and white British people. We will include analysis by ethnic subgroups where numbers allow. Across the four study sites, we expect to identify approximately 70,000 people from ethnic minorities and 480,000 white British people who have sought emergency care for injury. This will give ample power to undertake meaningful comparisons across aspects of presentation (e.g., proportion presenting with a specific condition), disposition (e.g., proportion admitted to hospital; length of stay) and further outcomes (e.g., re-attendance rates, mortality) over time and between cohorts and pre-specified subgroups.

Limitations in routine data will define a third study cohort, comprised of people presenting with injury but for whom no useable data on ethnicity are available. We will describe the characteristics and outcomes (e.g., age, sex, injury type and severity; and health outcomes) for this cohort and compare them with the people from ethnic minorities and white British cohorts. This will address our objective related to the quality of ethnicity data in emergency care settings.

WP3: Questionnaire survey

We will collate questionnaire data on a secure platform; initial processing will include data validation, assessment of its quality and completeness, and implementation of published

For peer review only - http://bmjopen.bmj.com/site/about/guidelines.xhtml

scoring algorithms. In our analyses we will report descriptive summaries of responses (using standard methods, including tabulated counts and percentages); comparative analyses, combining questionnaire outcomes with pre-specified factors and covariates; and, where feasible, description and comparison of questionnaire respondents and non-respondents.

WP4: Qualitative interviews and focus groups

We will use framework analysis [52] to analyse qualitative interview and focus group data. We will identify themes from our study questions, the literature and initial analysis of survey data to develop our framework. We will code transcripts according to these themes and refine as analysis progresses. Experienced qualitative researchers will lead analysis of interview and focus group transcripts. Two public contributors will help to validate the analysis process, supporting key stages of coding, refining themes and provide a critical stance [53]. We will use NVivo 11, computer assisted qualitative data analysis, to manage data. We will remove all identifiable data from interview and focus groups transcripts and assign a participant number for identification. Where appropriate, anonymous coded excerpts or quotes will be included in outputs.

WP5: Synthesis of quantitative and qualitative findings

We will synthesise and report on quantitative and qualitative findings by identifying meta-themes that cut across each component of the study [54]. We will interpret the results and consider similarities and differences, including recurring themes and issues that emerge from the scoping review, routine data, survey responses and people's views and experiences of injury and care received. We will use this evidence to inform our policy recommendations for improving injury care for people from ethnic minorities, including direction of future research.

For peer review only - http://bmjopen.bmj.com/site/about/guidelines.xhtml

ETHICS AND DISSEMINATION

We have obtained a favourable ethical approval from the Wales Research Ethics Committee (REC) (305391). We have also completed all necessary research permissions through the Health Research Authority (HRA). In addition, we obtained information governance approvals from the Confidentiality Advisory Group (CAG) to conduct data linkage and retrieval of outcomes for analysis from NHS Digital in England (via its Data Access Request Service (DARS)) and are in the process of gaining approval from eDRIS in Scotland (University of Edinburgh and NHS Lothian). Due to data protection and patient confidentiality, participating Trusts are unable to share medical records with peer researchers or Third Sector organisations. Therefore, the research paramedics and nurses will identify and recruit participants from routine records to take part in the questionnaire survey (WP3) and qualitative interviews (WP4).

Patient And Public Involvement

We will ensure our public contributors are actively involved in all aspects of the study [55]. We have strong relationships with people from ethnic minorities who have contributed experience-based expertise throughout the process of planning this proposal. We have drawn on the experiences and knowledge of two experienced public contributors to design the study who will join the Research Management Group to implement the research (TH, SS). We will recruit two additional public contributors to join the independent Study Steering Committee alongside clinical, policy, academic, methodological and subject experts. We will also regularly present progress and emerging findings of our study to two public advisory groups, the PRIME SUPER Group [56] and the SAIL Consumer Panel [57]. We will provide honoraria, briefings and other support as needed in line with best practice and report public involvement in our outputs [58].

For peer review only - http://bmjopen.bmj.com/site/about/guidelines.xhtml

Dissemination

We will include engagement with patient and professional groups, NHS managers, commissioners and policy makers and third sector organisations in our communication, publication and dissemination plan. We will use the plan to guide our second Stakeholder Event, which will take place once the study data collection and analysis are complete. The Stakeholder Event will be designed to be inclusive allowing patients, public contributors, third sector organisations, service providers and policy makers the space to share their views. At the event, we will discuss and refine our findings to ensure our results are credible and are widely shared with the community and service providers.

DISCUSSION

This is the first study in the UK to use routine anonymised linked data to compare outcomes and experiences for people from ethnic minorities and white British people when they present with injury to emergency health services. Our mixed-methods design builds on this innovative approach to capturing data by employing qualitative methods (WP4) to gain an in-depth understanding of a range of experiences, outcomes, and views about emergency care that are not available in routine health records. Our focus groups with asylum seekers and refugees (WP4) provide a valuable insight to the ways an already vulnerable population accesses and navigates emergency health services when contacted for care following injury.

The strength of this study lies in its multifaceted approach to study design, data collection and analysis which stems from the diverse study team. Collaborating with ambulance services, EDs, community members and third sector organisations not only strengthens the implementation of the study's research activities, but also ensures that the contribution this study makes to the evidence base will be informed by those who deliver and use emergency services.

For peer review only - http://bmjopen.bmj.com/site/about/guidelines.xhtml

FIGURES

Figure 1. Study design and participant recruitment flowchart 7

Figure 2. WP2 – Data flow for routine linked data epidemiology of injury presentation, care delivery and outcomes 9

Figure 3. WP3 – Data flow for questionnaire survey 13

AUTHORS CONTRIBUTION

FBa drafted the manuscript with editorial input from all authors – FBe, BAE, SG, TH, AK, AJ, JPA, RAL, AP, ITR, SS, ANS, AW, JW, HS. The research idea was conceived by AK and HS and developed by all authors. All authors read and approved the final manuscript.

COMPETING INTERESTS STATEMENT

There are no competing interests.

FUNDING STATEMENT

This work was supported by National Institute for Health and Care Research grant number NIHR132744.

REFERENCES

1. Aldridge E, Sethi D, Yon Y. Injuries: a call for public health action in Europe Injuries: a call for public health action in Europe. An update using the 2015 WHO global health estimates. *WHO Regional Office for Europe.* 2015. https://www.euro.who.int/__data/assets/pdf_file/0018/348102/WH08_IJ_complete-web-version.pdf?ua=1. (Accessed 9 Jun 2022).

For peer review only - http://bmjopen.bmj.com/site/about/guidelines.xhtml

2. NICE. Unintentional Injuries: Prevention Strategies for under 15s, in *Public Health Guideline* [PH29]. 2010. https://www.nice.org.uk/guidance/ph29. (Accessed 9 Jun 2022).

3. NHS digital. CDS Type 011 – Emergency Care Data Set (ECDS) User guide. *NHS Digital*. 2020. https://digital.nhs.uk/data-and-information/data-collections-and-datasets/data-sets/emergency-care-data-set-ecds/user-guide/data-group-injury-characteristics?key=#5-14-1-definition-group-status-and-justification-for-data-group (Accessed 9 Jun 2022).

4. Matheson J, Patterson J, Neilson L. Tackling Causes and Consequences of Health Inequalities: A Practical Guide. London: CRC Press 2020.

5. Public Health England. Public Health Outcomes Framework: Health Equity Report - Focus on ethnicity. 2017.

6. NHS. The NHS long term plan. https://www.longtermplan.nhs.uk/. (Accessed 9 Jun 2022).

7. Honeyford K, Coughlan C, Nijman RG, Expert P, Burcea G, Maconochie I, Kinderlerer A, Cooke GS, Costelloe CE. Changes in Emergency Department Activity and the First COVID-19 Lockdown: A Cross-sectional Study. *West J Emerg Med* 2021;22(3)603–607. DOI: 10.5811/westjem.2021.2.49614

8. Phung VH, Siriwardena N, Windle K, et al. Ethnicity and prehospital emergency care provided by ambulance services. *Race Equality Foundation* 2015 http://eprints.lincoln.ac.uk/id/eprint/17521/1/Health%20Briefing%2037.pdf. (Accessed 9 Jun 2022).

9. Khanom A, Alanazy W, Snooks H, et al. Asylum seekers' and refugees' experiences of accessing health care: a qualitative study. *BJGP Open* 2021;14;5(6):DOI:10.3399/BJGPO .2021.0059.

10. Graetz V, Rechel B, Groot W, et al. Utilization of health care services by migrants in Europe-a systematic literature review. *Br Med Bull* 2017;121(1):5-18.

For peer review only - http://bmjopen.bmj.com/site/about/guidelines.xhtml

11. Gunst M, Jarman K, Yarwood V, Rokadiya S, Capsaskis L, Orcutt M, Abbara A. Healthcare access for refugees in Greece: Challenges and opportunities. *Health Policy* 2019; 1;123(9):818-24 DOI:10.1016/j.healthpol.2019.06.003

12. Satinsky E, Fuhr DC, Woodward A, Sondorp E, Roberts B. Mental health care utilisation and access among refugees and asylum seekers in Europe: a systematic review. *Health Policy* 2019;1;123(9):851-63 DOI: 10.1016/j.healthpol.2019.02.007

13. Hewes H, Dai M, Mann NC, et al. Prehospital Pain Management: Disparity By Age and Race. *Prehosp Emerg Care* 2018; 22(2):189-197. DOI: 10.1080/10903127.2017. 1367444

14. Herrera-Escobar JP, Rivero R, Apoj M, et al. Long-term social dysfunction after trauma: What is the prevalence, risk factors, and associated outcomes? *Surgery* 2019;166(3):392-397.

15. Ali I, Vattigunta S, Jang JM, et al. Racial Disparities are Present in the Timing of Radiographic Assessment and Surgical Treatment of Hip Fractures. *Clin Orthop Relat Res* 2020;478(3):455-461.

16. LaPlant MB, Hess DJ. A review of racial/ethnic disparities in paediatric trauma care, treatment, and outcomes. *Trauma Surg Acute Care Open* 2019;86(3):540-550.

17. Hsia RY, Markowitz AJ, Lin F, et al. Ten-year trends in traumatic brain injury: a retrospective cohort study of California emergency department and hospital revisits and readmissions. *BMJ Open* 2018;8(12): e022297.

18. Haider AH, Chang Dc, Efron ER, et al. Race and insurance status as risk factors for trauma mortality. *Arch Surg* 2008;143(10):945-949

19. Athavale AM, Fu CY, Bokhari F, et al. Incidence of, Risk Factors for, and Mortality Associated With Severe Acute Kidney Injury After Gunshot Wound. *JAMA Netw open* 2019;2(12): e1917254,DOIi:10.1001/jamanetworkopen.2019.17254

For peer review only - http://bmjopen.bmj.com/site/about/guidelines.xhtml

20. Meixner C, Loder RT. The Demographics of Fractures and Dislocations Across the Entire United States due to Common Sports and Recreational Activities. *Sports health* 2020; 12(2): 159-169.

21. de Angelis P, Kaufman EJ, Barie PS, et al. Disparities in Timing of Trauma Consultation: A Trauma Registry Analysis of Patient and Injury Factors. *Journal Surg Res* 2019;242:357-362.

22. Roudsari B, Caetano, R, Field, C. Alcohol intoxication/dependence, ethnicity and utilisation of health care resources in a level I trauma center. *Injury* 2011;42(1):66-71.

23. Plener PL, Muns LM, Allroggen M, et al. Immigration as risk factor for non-suicidal self-injury and suicide attempts in adolescents in Germany. *Child Adolesc Psychiatry Ment Health* 2015;9(1):34.

24. Sakran JV, Nance M, Rail T, et al. Paediatric Firearm Injuries and Fatalities: Do Racial Disparities Exist? *Ann Surg* 2020;272(4):556-561.

25. Rojas-Velasquez, DA, Pluhar EL, Burns PA, et al. Nonsuicidal Self-Injury Among African American and Hispanic Adolescents and Young Adults: a Systematic Review. *Prev Sci* 2020;22(3):367-377.

26. Hodes D, O'Donnell NA, Pall K, et al. Epidemiological surveillance study of female genital mutilation in the UK. *Arch Dis Child* 2020;106(4)372-376.

27. Cézard G., Gruer L, Steiner M, et al. Ethnic variations in falls and road traffic injuries resulting in hospitalisation or death in Scotland: the Scottish Health and Ethnicity Linkage Study. *Public health* 2020;182:32-38.

28. Dean E, Taylor MJ, Francis H, et al. An exploration of community and culture related fire injury risks. *UKAIS* 2016, http://researchonline.ljmu.ac.uk/id/eprint/3523/

29. Loder RT, Robinson, TP. The demographics of patients presenting for sexual assault to US emergency departments. *J Forensic Leg Med* 2020;69:101887.

For peer review only - http://bmjopen.bmj.com/site/about/guidelines.xhtml

30. Steinbach R, Edwards P, Green J. Controlling for exposure changes the relationship between ethnicity, deprivation and injury: an observational study of child pedestrian injury rates in London. *Inj Prev* 2014;20(3):159-166. doi:10.1136/injuryprev-2012-040741

31. Sewell T, Aderin-Pocock M, Chughtai A, et al. The report of the Commission on Race and Ethnic Disparities 2021. https://www.gov.uk/government/publications/the-report-of-the-commission-on-race-and-ethnic-disparities

32. Asghar Z., Phung VH., Siriwardena, AN. Ethnicity and pre-hospital care for people with suspected cardiac pain: cross-sectional study. *J Eval Clin Pract* 2016;22(5):721-725.

33. Gill PS, Beavan J, Calvert M, et al. The unmet need for interpreting provision in UK primary care. *PLoS one* 2011;13;6(6):e20837, DOI: 10.1371/journal.pone.0020837

34. Kamal A, Hodson A, Pearce JM. A rapid systematic review of factors influencing COVID-19 vaccination uptake in minority ethnic groups in the UK. *Vaccines* 2021;1;9(10):1121. DOI: org/10.3390/vaccines9101121

35. Memon A, Taylor K, Mohebati LM, et al. Perceived barriers to accessing mental health services among black and minority ethnic (BME) communities: a qualitative study in Southeast England. *BMJ open* 2016;1;6(11):e012337, DOI: 10.1136/bmjopen-2016-012337

36. Goodacre S, Thomas B, Lee E, et al. Characterisation of 22445 patients attending UK emergency departments with suspected COVID-19 infection: Observational cohort study. *PLoS One* 2020;15(11):e0240206.

37. Creswell JW, Plano Clark VL. Chapter 3: Choosing a mixed methods design. In Creswell JW, Clark VL, eds. Designing and conducting mixed methods research. London: SAGE. 2011;(2)53-106.

38. Giddens, A. The constitution of society: Outline of the theory of structuration. *University of California Press;* 1984.

For peer review only - http://bmjopen.bmj.com/site/about/guidelines.xhtml

39. Ray V. A Theory of Racialized Organizations. *Am Sociol Rev* 2019;84(1):26-53. DOI: 10.1177/0003122418822335

40. Peters MD, Godfrey CM, Khalil H, et al. Guidance for conducting systematic scoping reviews. *Int J Evid Based Healthc* 2015;13(3):141-6. DOI: 10.1097/XEB.0000000000 000050.

41. Tricco AC, Lillie E, Zarin W, et al. PRISMA Extension for Scoping Reviews (PRISMAScR): Checklist and Explanation. *Ann Intern Med* 2018;169:467–473. DOI: 10.7326/M18-0850.

42. Whiting P, Rutjes AWS, Reitsma JB, et al. The development of QUADAS: a tool for the quality assessment of studies of diagnostic accuracy included in systematic reviews. *BMC Med Res Methodol* 2003;3:25 DOI:10.1186/1471-2288-3-25.

43. Mourby MJ, Doide KH, Jones, et al. Health Data Linkage for UK Public Interest Research: Key Obstacles and Solutions. *Int J Popul Data Sci* 2019;4(1):1093-1093.

44. Lyons RA, Jones KH, John G, et al. The SAIL databank: linking multiple health and social care datasets. *BMC Med Inform Decis Mak* 2009;9(1):1-8.

45. Piel FB, Parkers BL, Daby H, et al. The challenge of opt-outs from NHS data: a small-area perspective. *J Public Health (Oxf)* 2018;40(4):e594-e600.

46. Aspinall PJ. The utility and validity for public health of ethnicity categorization in the 1991, 2001 and 2011 British Censuses. *Public Health* 2011;125(10):680-687.

47. Edwards PJ, Roberts I, Clarke MK, et al. Methods to increase response to postal and electronic questionnaires. *Cochrane database of systematic reviews* 2009(3):MR000008.

48. Carey RG, Seibert JH. A patient survey system to measure quality improvement: questionnaire reliability and validity. *Med Care* 1993:834-845.

49. Jenkinson C, Chandola T, Coulter A, et al. An assessment of the construct validity of the SF-12 summary scores across ethnic groups. *J Public Health Med* 2001;1;23(3):187-94: DOI: 10.1093/pubmed/23.3.187

For peer review only - http://bmjopen.bmj.com/site/about/guidelines.xhtml

50. Salway S, Chowbey P, Such E, et al. Researching health inequalities with Community Researchers: practical, methodological and ethical challenges of an ‘inclusive’ research approach. *Res Involv Engagem* 2015;1(9) DOI: 10.1186/s40900-015-0009-4

51. Evans BA, Bedson E, Bell P, et al. Involving service users in trials: developing a standard operating procedure. *Trials* 2013;14(1):1-7.

52. Ziebland S, McPherson A. Making sense of qualitative data analysis: an introduction with illustrations from DIPEx (personal experiences of health and illness). *Med Educ* 2006;40(5):405-414.

53. Frost J, Gibson A, Harris-Golesworthy F, et al. Patient involvement in qualitative data analysis in a trial of a patient-centred intervention: reconciling lay knowledge and scientific method. *Health Expec.* 2018;21(6):1111-21. DOI:10.1111/hex.12814

54. O’Cathain A, Murphy E, Nicholl J. Three techniques for integrating data in mixed methods studies. *BMJ* 2010;17;341. DOI: 10.1136/bmj.c4587

55. Bovaird, T. Beyond engagement and participation: User and community coproduction of public services. *Public Adm Rev* 2007;67(5):846-860.

56. Evans BA, Gallanders J, Griffiths L, et al. Public involvement and engagement in primary and emergency care research: the story from PRIME Centre Wales. *IJPDS.* 2020;5(3):1363. DOI: 10.23889/ijpds.v5i3.1363

57. Jones KH, McNerney CL, Ford DV. Involving consumers in the work of a data linkage research unit. *Int J Consum Stud* 2013;38(1)45-51DOI:10.1111/ijcs.12062

58. Staniszewska S, Brett J, Simera I, et al. GRIPP2 reporting checklists: tools to improve reporting of patient and public involvement in research BMJ 2017; 358 DOI: 10.1136/bmj.j3453

For peer review only

For peer review only - http://bmjopen.bmj.com/site/about/guidelines.xhtml

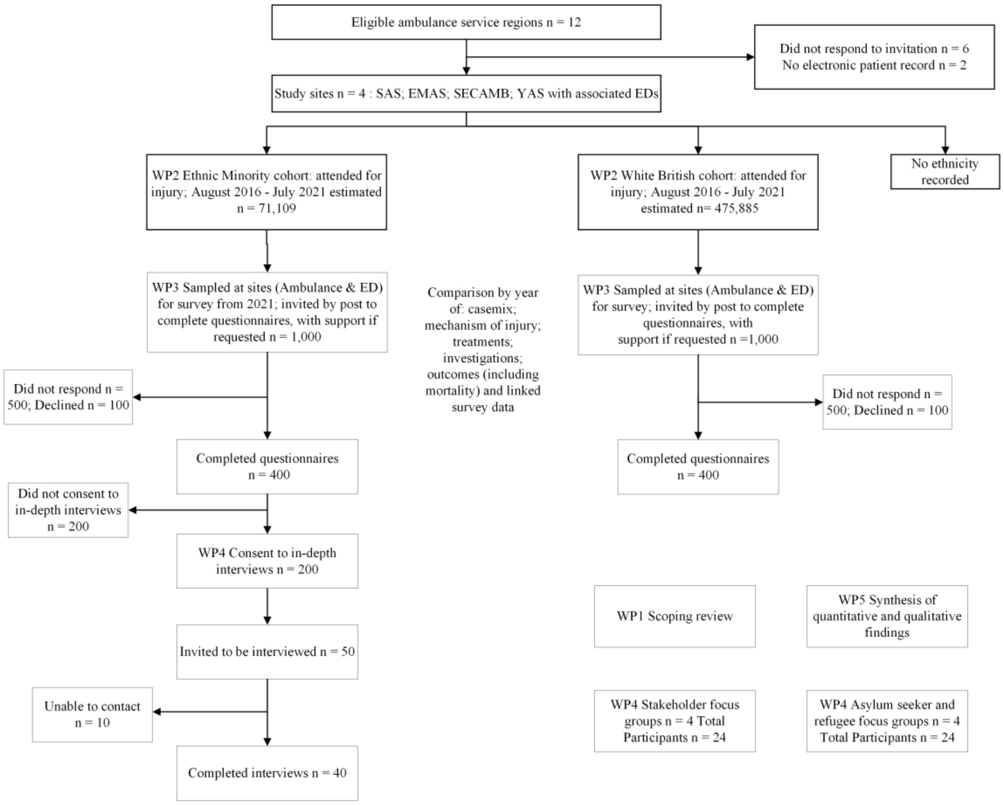

Figure 1. Study design and participant recruitment flowchart

309x246mm (300 x 300 DPI)

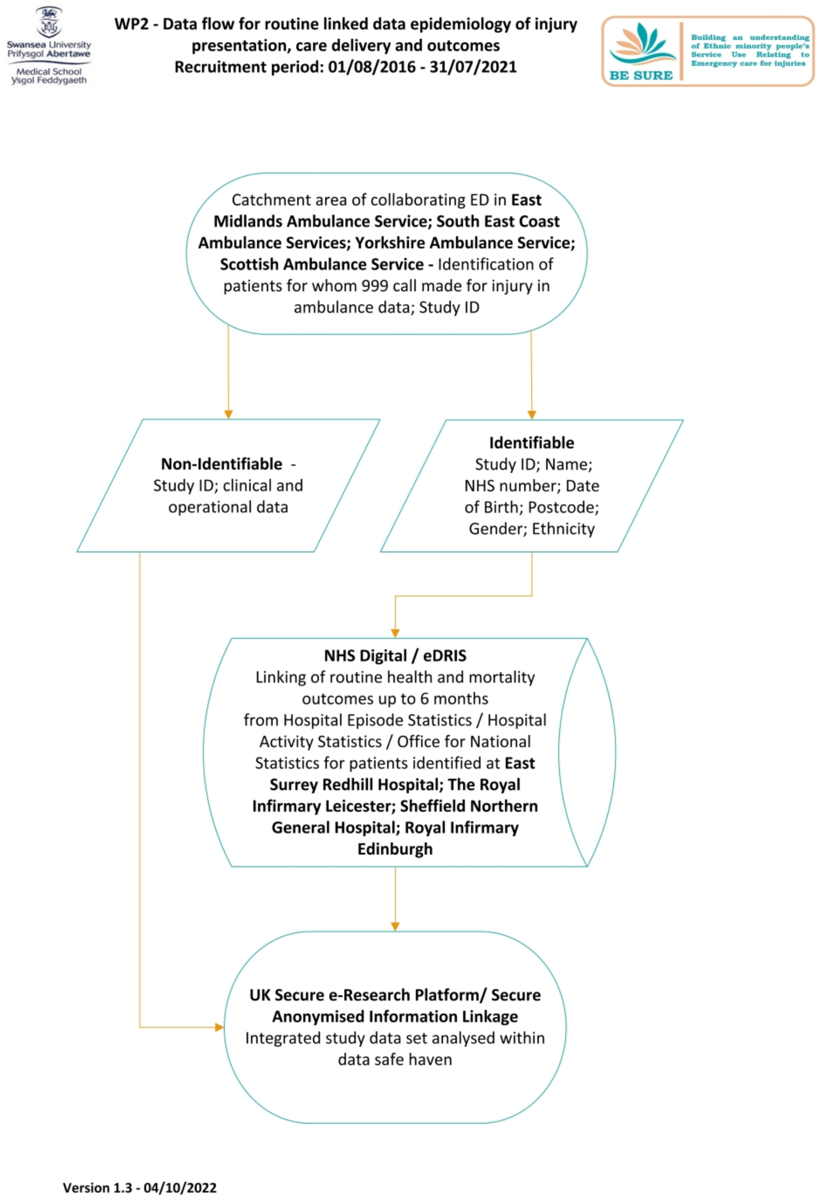

Figure 2. WP2 – Data flow for routine linked data epidemiology of injury presentation, care delivery and outcomes

319x468mm (300 x 300 DPI)

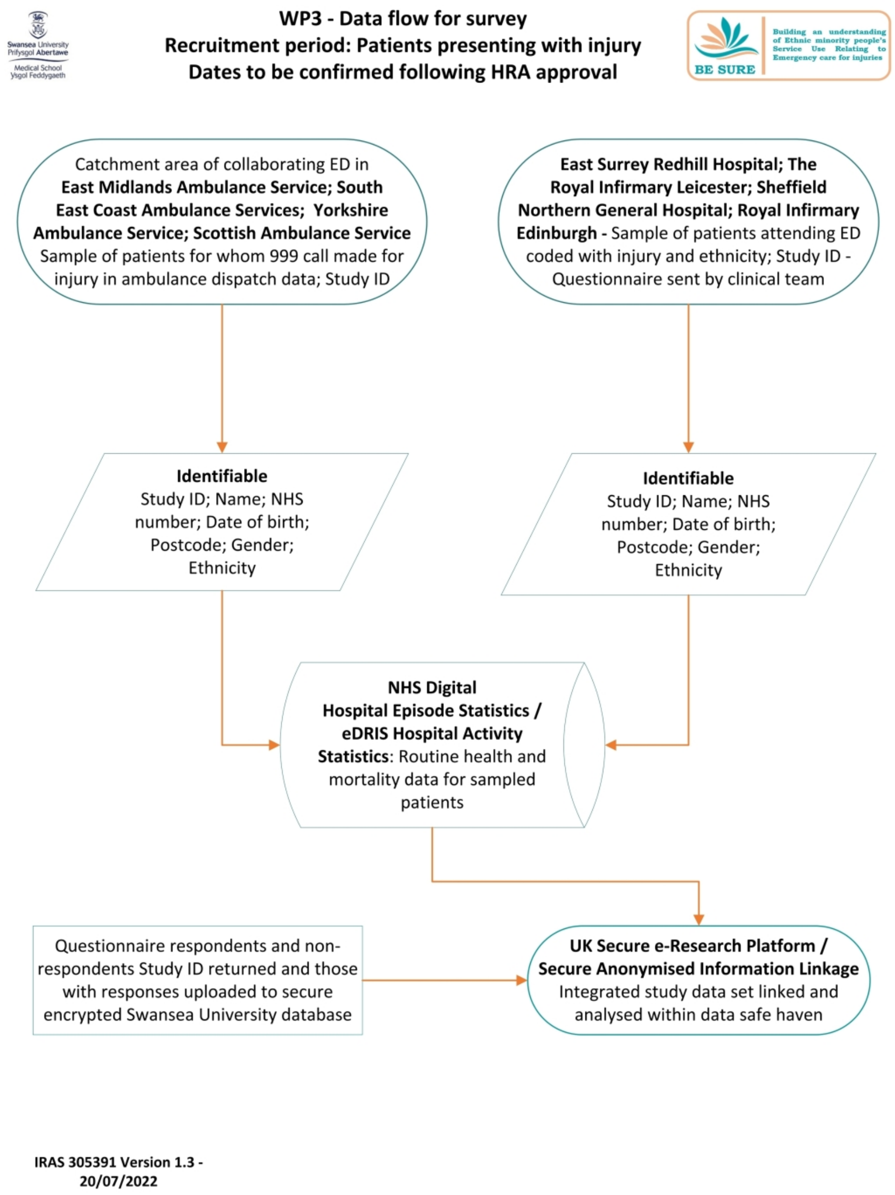

Figure 3. WP3 – Data flow for questionnaire survey

239x322mm (300 x 300 DPI)

BE SURE – Scoping Review Database Search Strategy

Databases Searched: EBSCO = CINAHL, MEDLINE AND PsycInfo; SCOPUS and COCHRANE

Limits applied – Date: January 2010 – May 2022; Human (only applied in EBSCO)

Search terms 1-5 combined with AND

1. **Population**:

(Ethnicit* OR Race OR "BAME" OR "Black, Asian and minorit* ethnic*" OR "Black, Asian & minorit* ethnic*" OR Immigrant* OR Migrant* OR "Asylum seeker*" OR Refugee* OR Multicultural OR Language OR English OR Ethnic OR Religion OR Culture OR Latin*o OR Latina OR Indigenous OR Aboriginal OR Native OR "First Nation*" OR "Afro-Caribbean*" OR "African American*" OR Gyps* OR Roma OR "Hispanic American")

2. **Emergency Service:**

("emergency service*" OR ambulance* OR "Emergency department*" OR "Accident and emergency department*" OR "Accident & emergency department*" OR A&E OR "emergency room*" OR "emergency care*" OR "999 calls and admission*" OR "Emergency health service*" OR 999 OR 911 OR "Ambulance service" OR "Ambulance trust" OR "Emergency medical service*" OR "EMS" OR "Patient transport" OR "control room")

3. **Patient Category:**

(Patient OR Inpatient OR Outpatient OR "In-Patient" OR "Out-Patient")

4. **Clinical care:**

("Quality of care" OR Pain OR "Pain management" OR "length of hospital stay*" OR disability OR "Process of care" OR "Repeat attendance*" OR "Mortality" OR Treatment OR "Emergency treatment" OR "Clinical care" OR Rehabilitation)

5. **Injury:**

(Injur* OR assault* OR "Domestic violence" OR "Intimate partner violence" OR "self-harm" OR suicide OR accident* OR Violence)

For peer review only - http://bmjopen.bmj.com/site/about/guidelines.xhtml

For peer review only

BE SURE

NIHR | National Institute for Health Research

Swansea University Prifysgol Abertawe Medical School Ysgol Feddygaeth

<<Study ID>>

A. Your Details

1. What is your gender ☐ Female ☐ Male ☐ Other ☐ Prefer not to say

2. What is your age in years? ☐

3. What ethnicity do you identify as?

☐ White British	☐ White/Asian	Caribbean
☐ White Irish	☐ Other mixed	African
☐ Other White	Indian	Other Black
☐ White/Black Caribbean	Pakistani	Chinese
☐ White/Black African	Bangladeshi	Other ethnic group? Please
☐	Other Asian	Specify ☐

☐ 4. What is your preferred language?

☐ English	☐ Bengali	☐ Mandarin	☐ Slavak
☐ Scots	☐ Gujarati	☐ Cantonese	Somali
☐ Polish	☐ Arabic	☐ Spanish	Lithuanian ☐
☐ Punjabi	☐ French	☐ Portuguese	German
☐ Urdu	☐	☐ Tamil	Persian/Farsi
☐	☐	☐ Turkish	Tagalog/Filipino
☐	☐	☐	Romanian
	☐	☐	Other

For peer review only - http://bmjopen.bmj.com/site/about/guidelines.xhtml

BE SURE NIHR | National Institute for Health Research Swansea University Prifysgol Abertawe Medical School Ysgol Feddygaeth

<<Study ID>>

5. What do you feel is your level of English?

Please tick all that apply.

5.1. Reading.

- ☐ I can read English well
- ☐ I can read a little English
- ☐ I cannot read English

5.2. Speaking.

- ☐ I can speak English well
- ☐ I can speak a little English
- ☐ I cannot speak English

5.3. Holding a conversation.

- ☐ I can talk in English with a health professional (i.e. doctor, nurse, or paramedic)
- ☐ I find it difficult to talk in English with a health professional
- ☐ I cannot talk in English with a health professional

6. How long have you lived in the UK?

- ☐ My whole life, go to question 7
- ☐ More than 5 years
- ☐ 1-5 years
- ☐ Less than 1 year

6.1. What country were you living in before settling in the UK?

☐

B. Your knowledge of healthcare services

In this section we want to understand what you know about the different emergency healthcare services that are available.

7. Do you know healthcare care is free in the United Kingdom?

☐ Yes ☐ No

For peer review only - http://bmjopen.bmj.com/site/about/guidelines.xhtml

BE SURE NIHR | National Institute for Health Research Swansea University Prifysgol Abertawe Medical School Ysgol Feddygaeth

<<Study ID>>

8. Do you know 999 ambulance service care is free in the United Kingdom?

☐ Yes ☐ No ☐ Not Sure

9. Do you know Emergency Department care is free in the United Kingdom?

☐ Yes ☐ No ☐ Not Sure

10. Have you heard of the following services?

	Not heard of the service	Heard of the service but don't know how to contact	Have heard of and would know how to access the service
NHS 111/NHS Direct telephone advice			
Minor Injuries Unit			
999 ambulance service			
Emergency Department (A&E)			

11. Did you know that the NHS should provide you with an interpreter if you need one?

☐ Yes ☐ No ☐ Not Sure

C. Who you contact when hurt

12. How often have you called the 999 ambulance service in the last 12 months for an injury?

☐ 0 ☐ 1-2 ☐ 2-5 ☐ 5+

For peer review only - http://bmjopen.bmj.com/site/about/guidelines.xhtml

BE SURE | NIHR | National Institute for Health Research | Swansea University Prifysgol Abertawe Medical School Ysgol Feddygaeth

<<Study ID>>

13. How often have you visited an Emergency Department in the last 12 months for an injury?

☐ 0 ☐ 1-2 ☐ 2-5 ☐ 5+

D. Your experience of care

In this section we want to understand your experience of healthcare provided by the 999 ambulance service, Emergency Department, or both on the date on the cover letter of this questionnaire. You only need to answer questions that are relevant to your experience.

Calling 999 ambulance service

14. Did you make the 999 ambulance service call?

☐ Yes ☐ No if no – go to Q.27, Page 5.

15. Do you feel that the 999 ambulance service call taker listened to you?

☐ Yes, completely ☐ Yes, somewhat ☐ No ☐ Don't know

16. Did you think that the 999 ambulance service call taker asked questions relevant to your problem?

☐ Yes, completely ☐ Yes, somewhat ☐ No ☐ Don't know

17. Did you feel reassured when speaking to the 999 ambulance service call taker?

☐ Yes, completely ☐ Yes, somewhat ☐ No ☐ Don't know

For peer review only - http://bmjopen.bmj.com/site/about/guidelines.xhtml

BE SURE | NIHR | National Institute for Health Research | Swansea University Prifysgol Abertawe Medical School Ysgol Feddygaeth

<<Study ID>>

18. Did the 999 ambulance service call taker tell you an ambulance would be sent to you?

☐ Yes

☐ No

If no, what did the call taker advise? Tick all that apply and go to Q.27, Page 7

☐ Manage the problem at home yourself
☐ Go to the Pharmacist
☐ Go to your GP
☐ Call NHS 111
☐ Call back if the problem gets worse

19. If you needed help, did the 999 ambulance service call taker tell you how long you could expect to wait for help to arrive?

☐ Yes, but wait was shorter
☐ Yes, and that was about right
☐ Yes, but wait was longer
☐ No
☐ Don't know

20. How would you rate your experience of using 999 ambulance service telephone service on this occasion?

☐ Very good
☐ Good
☐ Fair
☐ Poor
☐ Very Poor

When the Ambulance Arrived

21. Did the paramedics or ambulance workers you saw explain what they were doing in a way you could understand?

☐ Yes, completely
☐ Yes, somewhat
☐ No
☐ Don't know

For peer review only - http://bmjopen.bmj.com/site/about/guidelines.xhtml

BE SURE | NIHR National Institute for Health Research | Swansea University Prifysgol Abertawe Medical School Ysgol Feddygaeth

<<Study ID>>

22. Did the paramedics or ambulance workers explain what was wrong with you in a way you could understand?

☐ Yes, completely ☐ Yes, somewhat ☐ No ☐ Don't know

23. If you were in pain, did the paramedics or ambulance workers do all they could to help?

☐ Yes, definitely ☐ Yes, somewhat ☐ No ☐ I was not in any pain

24. Once the paramedics or ambulance workers completed their work, did they tell you what to do if you continued to feel unwell or if your condition worsened?

☐ Yes, they told me to call the ambulance service back ☐ Yes, they told me to call my GP ☐ Yes, they told me to call another health care professional ☐ Yes, they gave me an information leaflet ☐ No, they did not provide any advice if I continued to feel unwell ☐ No advice was wanted/ needed ☐ Don't know

25. Overall, how well do you think you were looked after by the paramedics or ambulance workers?

☐ Very well ☐ Fairly well ☐ Not very well ☐ Not at all well

26. Did you travel to the Emergency Department in the ambulance?

☐ Yes ☐ No – if no, got to Q32, Page 8.

Journey to Emergency Department

27. Was it your decision to go to the Emergency Department?

For peer review only - http://bmjopen.bmj.com/site/about/guidelines.xhtml

BE SURE

NIHR | National Institute for Health Research

Swansea University Prifysgol Abertawe Medical School Ysgol Feddygaeth

<<Study ID>>

☐ Yes ☐ ☐ No Not sure

28. Was the length of time the ambulance workers spent with you before taking you to hospital or elsewhere what you expected?

☐ Yes ☐ No, it was too long ☐ No, it was too short ☐ Don't know

29. Did the ambulance workers talk to you during the journey? (Please tick all that apply)

☐ Yes, they told me what was happening

☐ Yes, they asked me about how I was feeling

☐ Yes, they talked to me about what would happen when we got to the hospital or elsewhere

☐ Yes, they talked about something else

☐ No, they didn't talk to me

30. How well do you feel you understood what the paramedics or ambulance workers were saying to you during your journey?

☐ Very well ☐ Well ☐ Neutral ☐ Not well ☐ Not at all

31. When you arrived at the hospital did the paramedics or ambulance workers wait with you until you were seen by a doctor or nurse?

☐ Yes ☐ No ☐ Can't remember

Go to Q.41, Page 9.

For peer review only - http://bmjopen.bmj.com/site/about/guidelines.xhtml

BE SURE | NIHR National Institute for Health Research | Swansea University Prifysgol Abertawe Medical School Ysgol Feddygaeth

<<Study ID>>

Arriving at the Emergency Department yourself

Please complete this section only if you travelled to the Emergency Department without an ambulance; if an ambulance brought you to the Emergency Department - go to Q.41, Page 9.

32. Who did you go to the Emergency Department with?

☐ Myself ☐ Neighbour ☐ Health professional
☐ Friend Family member Other (specify)
☐ Care worker

33. When you arrived at the Emergency Department, was it clear to you where to register?

☐ Yes, definitely ☐ Yes, somewhat ☐ No, this was not needed ☐ No, this would have helped ☐ Can't remember

34. Did anyone help to explain your problem to the person who

☐ Friend ☐ Care Worker ☐ Not Applicable
☐ Neighbour ☐ Family ☐ Other (please specify)
☐ Another patient in Emergency Department registered you?

35. Did the person who registered you understand your problem?

☐ Yes, completely ☐ Yes, somewhat ☐ No ☐ Don't know ☐ Not Applicable

36. After your registration, did you have to wait to be seen again?

☐ Yes ☐ No ☐ Can't remember (If no or can't remember, go to Q.41, Page 9.)

☐ ☐ ☐ ☐ ☐

For peer review only - http://bmjopen.bmj.com/site/about/guidelines.xhtml

BE SURE | NIHR National Institute for Health Research | Swansea University Prifysgol Abertawe Medical School Ysgol Feddygaeth

<<Study ID>>

I did not have to wait at all	The wait was shorter than expected	The wait was about as long as expected	The wait was longer than expected	Don't Know

37. Was the length of time you waited to be seen again what you expected?

38. While you were waiting, did someone tell you what was happening?

- ☐ Yes, definitely
- ☐ Yes, somewhat
- ☐ No, this was not needed
- ☐ No, this would have helped
- ☐ Can't remember

39. While you were waiting, did someone tell you what to do?

- ☐ Yes, definitely
- ☐ Yes, somewhat
- ☐ No, this was not needed
- ☐ No, this would have helped
- ☐ Can't remember

40. While you were waiting, did you get all the things you needed (like medicine, food, drink and toilets)?

- ☐ Yes, definitely
- ☐ Yes, somewhat
- ☐ No
- ☐ I did not need anything
- ☐ Don't know

Receiving Care

41. Could you understand when Emergency Department staff explained...

41.1. What they were doing?

- ☐ Yes, completely
- ☐ Yes, somewhat
- ☐ No
- ☐ Don't know

41.2. What was wrong with you?

☐ ☐ ☐ ☐

For peer review only - http://bmjopen.bmj.com/site/about/guidelines.xhtml
For peer review only

BE SURE | NIHR National Institute for Health Research | Swansea University Prifysgol Abertawe Medical School Ysgol Feddygaeth

<<Study ID>>

Yes, completely | Yes, somewhat | No | Don't know

42. If you were in pain, did Emergency Department staff do all they could to help your pain?

☐ Yes, definitely ☐ Yes, somewhat ☐ No ☐ I was not in pain ☐ Don't know

43. Were you given enough privacy when being treated and examined?

☐ Yes, definitely ☐ Yes, somewhat ☐ No ☐ I did not want privacy ☐ Don't know

44. After your attendance at the Emergency Department, what happened?

☐ I was admitted to hospital

☐ I was told to go home

☐ I was told to go see another health care worker (please specify)

☐ I discharged myself and went..., (please specify where)

45. Did anyone at the Emergency Department tell you when you could restart your usual activities?

☐ Yes definitely ☐ Yes somewhat ☐ No ☐ This was not necessary ☐ Don't know

46. Did anyone at the Emergency Department tell you how to provide self-care at home after your hospital care?

☐ ☐ ☐ ☐ ☐

For peer review only - http://bmjopen.bmj.com/site/about/guidelines.xhtml

BE SURE

NIHR | National Institute for Health Research

Swansea University Prifysgol Abertawe
Medical School Ysgol Feddygaeth

<<Study ID>>

Yes definitely	Yes somewhat	No	This was not necessary	Don't know

47. Did anyone at the Emergency Department tell you what to do or who to contact if you were still worried about anything?

☐ Yes ☐ No ☐ Don't know

E. Satisfaction with Care

Satisfaction with 999 Ambulance Service

If you travelled to the Emergency Department without an ambulance - go to Q.50, Page 13.

48. Please read the statements below and place an **X** in the box that reflects your agreement with each.

	Not applicable	Strongly agree	Agree	Neither agree nor disagree	Disagree	Strongly disagree
The ambulance crew who attended me listened carefully to my problem						
I think the crew were polite						
I got the advice I needed						
I was reassured by the advice given to me						
I was satisfied with the explanation I was given						
I was given advice about when to get more help						

For peer review only - http://bmjopen.bmj.com/site/about/guidelines.xhtml

For peer review only

<<Study ID>>

I was satisfied with the ambulance crew						
I was made to feel I was wasting the crew's time						

49. If the same health problem arose again, would you call an ambulance to help you with your problem?

☐ Yes ☐ No ☐ Don't know

If no, who would you contact for advice or help, or would you look after yourself?

Tick any that apply.

☐ I would go to ED myself

☐ I would look after myself

☐

NHS Direct/NHS111

Careline

☐ GP

☐

☐

Mental Health Team

District Nurse

Social Worker

Other – please specify

☐

☐

☐

For peer review only

For peer review only - http://bmjopen.bmj.com/site/about/guidelines.xhtml

BE SURE

NIHR | National Institute for Health Research

Swansea University Prifysgol Abertawe
Medical School Ysgol Feddygaeth

<<Study ID>>

Satisfaction with Emergency Department (A&E)

50. Please read statements below and place an X in the box that reflects your agreement with each.

	Not applicable	Strongly agree	Agree	Neither agree nor disagree	Disagree	Strongly disagree
The Emergency Department staff who supported me listened carefully to my problem						
I think the staff were polite						
I got the amount of advice I needed						
I was reassured by the advice I received						
I was satisfied with the explanation I received						
I was given advice about when to get more help						
I was generally satisfied with the Emergency Department staff						
I was made to feel I was wasting the worker's time						

For peer review only - http://bmjopen.bmj.com/site/about/guidelines.xhtml

NIHR | National Institute for Health Research

BE SURE

Swansea University Prifysgol Abertawe Medical School Ysgol Feddygaeth

<<Study ID>>

51. If the same health problem arose again, would you go to the Emergency Department to help you with your problem?

☐ Yes ☐ No ☐ Don't know

If no, who would you contact for advice or help, or would you treat/look after yourself?

Tick any that apply.

☐ I would call 999	☐ GP	☐ Social Worker
☐ I would look after myself	☐ Mental Health Team	☐ Careline
☐ NHS Direct/NHS 111	☐ District Nurse	☐ Other – please specify

F. Your health and wellbeing

This section asks for your views about your health and how well you are able to do your usual activities. **Answer each question by choosing just one answer**. If you are unsure how to answer a question, please give the best answer you can.

1. **In general, would you say your health is:**

☐	☐	☐	☐	☐
Excellent	Very Good	Good	Fair	Poor

The following questions are about activities you might do during a typical day. Does your health now limit you in these activities? If so, how much?

	YES, limited a lot	YES, limited a little	NO, not limited at all
2. **Moderate activities such as** moving table, pushing vacuum cleaner, bowling, or playing golf.	☐	☐	☐
3. Climbing **several** flights of stairs.	☐	☐	☐

For peer review only - http://bmjopen.bmj.com/site/about/guidelines.xhtml

BE SURE

NIHR | National Institute for Health Research

Swansea University Prifysgol Abertawe Medical School Ysgol Feddygaeth

<<Study ID>>

During the <u>past 4 weeks</u>, have you had any of the following problems with your work or other daily activities <u>as a result of your physical health</u>?

	YES	NO
4. **Accomplished less** than you would like.	☐	☐
5. Were limited in the **kind** of work or other activities.	☐	☐

During the <u>past 4 weeks</u>, have you had any of the following problems with your work or other daily activities <u>as a result of any emotional problems</u> (like feeling anxious or depressed)?

	YES	NO
6. **Accomplished less** than you would like.	☐	☐
7. Did work or activities **less carefully than usual**.	☐	☐

8. **During the <u>past 4 weeks</u>, how much <u>did pain interfere</u> with your normal work (including work outside the home and housework)?**

☐	☐	☐	☐	☐
Not at all	A little	Moderately	A lot	Extremely

For peer review only - http://bmjopen.bmj.com/site/about/guidelines.xhtml

BE SURE | NIHR National Institute for Health Research | Swansea University Prifysgol Abertawe Medical School Ysgol Feddygaeth

<<Study ID>>

These questions are about how you have been feeling during the past 4 weeks.
For each question, please give the answer that comes closest to the way you have been feeling.
How much of the time during the past 4 weeks…

	All of the time	Most of the time	A good bit of the time	Some of the time	A little bit of the time	None of the time
9. Have you felt calm and peaceful?	☐	☐	☐	☐	☐	☐
10. Did you have a lot of energy?	☐	☐	☐	☐	☐	☐
11. Have you felt down-hearted and blue?	☐	☐	☐	☐	☐	☐

12. **During the past 4 weeks, how much of the time has your physical health or emotional problems interfered with your social activities (like visiting friends, relatives, etc.)?**

☐ All of the time ☐ Most of the time ☐ Some of the time ☐ A little of the time ☐ None of the time

G. Final questions and thank you

52. Please enter today's date.

53. Did anyone help you to complete this questionnaire (family, friend, support worker, or researcher)?

☐ Yes ☐ No

If yes, please describe your relationship with your helper.

For peer review only - http://bmjopen.bmj.com/site/about/guidelines.xhtml

NIHR | National Institute for Health Research

BE SURE

Swansea University Prifysgol Abertawe Medical School Ysgol Feddygaeth

<<Study ID>>

We would like to link your questionnaire to your routine NHS information anonymously. Place an **X** in the box if you **DO NOT** agree for us to link your questionnaire answers to your NHS information. ☐

54. We would like to invite you to take part in a one-to-one interview by telephone or in person to talk about the experience you described in this questionnaire. Would you be interested in talking to us about your experience of care?

☐ Yes ☐ No

If yes, please provide your contact details and we will arrange a convenient time and date for the interview.

Name	
Phone	
Email	

If you would like to receive a £10 High Street voucher for completing this questionnaire, please provide your full name and email address below. If you do not have an email address, please provide your full home address including post code.

Name	
Email	
Address and post code	

Thank you for your helping us with this study.

For peer review only - http://bmjopen.bmj.com/site/about/guidelines.xhtml

BE SURE Interview Guide

Interviews with people identifying from an ethnic minority

Aim: to give people an opportunity to provide their own narrative about their circumstances, routes to seeking care, injury experiences (personal or family), views regarding their need, service use, care received, and post injury impact on physical and mental health including terminology (labels) used.

Guide

We have contacted you because you indicated on our survey that you wanted to participate in an interview to talk about your experience and journey of care on [*insert date*]. We will focus on that experience in this interview, but we will also ask other questions about your general experience of any other times in the past when you had an injury, and your use of ambulance and emergency services.

1. Can you tell me what happened on that day?
   a) Injury to self or other?
   b) Nature of injury or incident how, what, when it happened? Who else was there?
   c) What help did ambulance and emergency care on this occasion?
2. Why did you make the above choice?
3. What was the experience like calling 999?

   E.g. Prompts for their journey of care – calling 999, talking to someone on the phone, waiting, ambulance attending, ambulance to the hospital, transfer, receiving care at the hospital, aftercare - ask about feelings of being discriminated against at each stage and what type of, based on, and how discrimination – language, ethnicity, religion, gender, sexuality, lass, geography, etc.

4. What was the experience like in the emergency department?

For peer review only - http://bmjopen.bmj.com/site/about/guidelines.xhtml

BE SURE Interview Guide

E.g. Prompts for their **journey of care** – triage, waiting, receiving care at the hospital, aftercare - ask about feelings of being discriminated against at each stage and what type of, based on, and how discrimination – language, ethnicity, religion, gender, sexuality, class, geography, etc.

5. Have you had any other experience of getting health care after an injury?

a) How did that time compare with the time we've been talking about?

6. If you were injured would you call 999 and/or go to the emergency department again?

a) What would you think about when you decided what to do?

b) Who is the emergency department for?

7. Can you think of any other ways to get help after an injury?

a) Prompts: NHS111, pharmacy, GP, family/friends

For peer review only

Preferred Reporting Items for Systematic reviews and Meta-Analyses extension for Scoping Reviews (PRISMA-ScR) Checklist

SECTION	ITEM	PRISMA-ScR CHECKLIST ITEM	REPORTED ON PAGE #
**TITLE**			
Title	1	Identify the report as a scoping review.	1
**ABSTRACT**			
Structured summary	2	Provide a structured summary that includes (as applicable): background, objectives, eligibility criteria, sources of evidence, charting methods, results, and conclusions that relate to the review questions and objectives.	2
**INTRODUCTION**			
Rationale	3	Describe the rationale for the review in the context of what is already known. Explain why the review questions/objectives lend themselves to a scoping review approach.	Not applicable
Objectives	4	Provide an explicit statement of the questions and objectives being addressed with reference to their key elements (e.g., population or participants, concepts, and context) or other relevant key elements used to conceptualize the review questions and/or objectives.	6
**METHODS**			
Protocol and registration	5	Indicate whether a review protocol exists; state if and where it can be accessed (e.g., a Web address); and if available, provide registration information, including the registration number.	Not applicable
Eligibility criteria	6	Specify characteristics of the sources of evidence used as eligibility criteria (e.g., years considered, language, and publication status), and provide a rationale.	Not applicable
Information sources*	7	Describe all information sources in the search (e.g., databases with dates of coverage and contact with authors to identify additional sources), as well as the date the most recent search was executed.	Not applicable
Search	8	Present the full electronic search strategy for at least 1 database, including any limits used, such that it could be repeated.	Not applicable
Selection of sources of evidence†	9	State the process for selecting sources of evidence (i.e., screening and eligibility) included in the scoping review.	Not applicable
Data charting process‡	10	Describe the methods of charting data from the included sources of evidence (e.g., calibrated forms or forms that have been tested by the team before their use, and whether data charting was done independently or in duplicate) and any processes for obtaining and confirming data from investigators.	Not applicable
Data items	11	List and define all variables for which data were sought and any assumptions and simplifications made.	Not applicable
Critical appraisal of individual sources of evidence§	12	If done, provide a rationale for conducting a critical appraisal of included sources of evidence; describe the methods used and how this information was used in any data synthesis (if appropriate).	Not applicable

St. Michael's
Inspired Care.
Inspiring Science.

For peer review only - http://bmjopen.bmj.com/site/about/guidelines.xhtml

SECTION	ITEM	PRISMA-ScR CHECKLIST ITEM	REPORTED ON PAGE #
Synthesis of results	13	Describe the methods of handling and summarizing the data that were charted.	Not applicable
**RESULTS**			
Selection of sources of evidence	14	Give numbers of sources of evidence screened, assessed for eligibility, and included in the review, with reasons for exclusions at each stage, ideally using a flow diagram.	Not applicable
Characteristics of sources of evidence	15	For each source of evidence, present characteristics for which data were charted and provide the citations.	Not applicable
Critical appraisal within sources of evidence	16	If done, present data on critical appraisal of included sources of evidence (see item 12).	Not applicable
Results of individual sources of evidence	17	For each included source of evidence, present the relevant data that were charted that relate to the review questions and objectives.	Not applicable
Synthesis of results	18	Summarize and/or present the charting results as they relate to the review questions and objectives.	Not applicable
**DISCUSSION**			
Summary of evidence	19	Summarize the main results (including an overview of concepts, themes, and types of evidence available), link to the review questions and objectives, and consider the relevance to key groups.	18
Limitations	20	Discuss the limitations of the scoping review process.	3
Conclusions	21	Provide a general interpretation of the results with respect to the review questions and objectives, as well as potential implications and/or next steps.	18
**FUNDING**			
Funding	22	Describe sources of funding for the included sources of evidence, as well as sources of funding for the scoping review. Describe the role of the funders of the scoping review.	19

JBI = Joanna Briggs Institute; PRISMA-ScR = Preferred Reporting Items for Systematic reviews and Meta-Analyses extension for Scoping Reviews.
* Where *sources of evidence* (see second footnote) are compiled from, such as bibliographic databases, social media platforms, and Web sites.
† A more inclusive/heterogeneous term used to account for the different types of evidence or data sources (e.g., quantitative and/or qualitative research, expert opinion, and policy documents) that may be eligible in a scoping review as opposed to only studies. This is not to be confused with *information sources* (see first footnote).
‡ The frameworks by Arksey and O'Malley (6) and Levac and colleagues (7) and the JBI guidance (4, 5) refer to the process of data extraction in a scoping review as data charting.
§ The process of systematically examining research evidence to assess its validity, results, and relevance before using it to inform a decision. This term is used for items 12 and 19 instead of "risk of bias" (which is more applicable to systematic reviews of interventions) to include and acknowledge the various sources of evidence that may be used in a scoping review (e.g., quantitative and/or qualitative research, expert opinion, and policy document).

From: Tricco AC, Lillie E, Zarin W, O'Brien KK, Colquhoun H, Levac D, et al. PRISMA Extension for Scoping Reviews (PRISMAScR): Checklist and Explanation. Ann Intern Med. 2018;169:467–473. doi: 10.7326/M18-0850.

For peer review only - http://bmjopen.bmj.com/site/about/guidelines.xhtml

TREND Statement Checklist

Paper Section/ Topic	Item No	Descriptor	Reported? ✓	Pg #
**Title and Abstract**				
Title and Abstract	1	• Information on how unit were allocated to interventions	✓	1
		• Structured abstract recommended	✓	1
		• Information on target population or study sample	✓	1
**Introduction**				
Background	2	• Scientific background and explanation of rationale	✓	3-4
		• Theories used in designing behavioral interventions	✓	6
**Methods**				
Participants	3	• Eligibility criteria for participants, including criteria at different levels in recruitment/sampling plan (e.g., cities, clinics, subjects)	✓	7
		Method of recruitment (e.g., referral, self-selection), including the sampling method if a systematic sampling plan was implemented	✓	8-14
		Recruitment setting	✓	5-6
		Settings and locations where the data were collected	✓	5-6
Interventions	4	• Details of the interventions intended for each study condition and how and when they were actually administered, specifically including:		
		○ Content: what was given?		
		○ Delivery method: how was the content given?		
		○ Unit of delivery: how were the subjects grouped during delivery?		
		○ Deliverer: who delivered the intervention?		
		○ Setting: where was the intervention delivered?		
		○ Exposure quantity and duration: how many sessions or episodes or events were intended to be delivered? How long were they intended to last?		
		○ Time span: how long was it intended to take to deliver the intervention to each unit?		
		○ Activities to increase compliance or adherence (e.g., incentives)		
Objectives	5	• Specific objectives and hypotheses	✓	4-5
Outcomes	6	• Clearly defined primary and secondary outcome measures	✓	5
		• Methods used to collect data and any methods used to enhance the quality of measurements	✓	6-7
		• Information on validated instruments such as psychometric and biometric properties		
Sample Size	7	How sample size was determined and, when applicable, explanation of any interim analyses and stopping rules	✓	5
Assignment Method	8	Unit of assignment (the unit being assigned to study condition, e.g., individual, group, community)		
		Method used to assign units to study conditions, including details of any restriction (e.g., blocking, stratification, minimization)		
		Inclusion of aspects employed to help minimize potential bias induced due to non-randomization (e.g., matching)		
Blinding (masking)	9	• Whether or not participants, those administering the interventions, and those assessing the outcomes were blinded to study condition assignment; if so, statement regarding how the blinding was accomplished and how it was assessed.		

For peer review only - http://bmjopen.bmj.com/site/about/guidelines.xhtml

TREND Statement Checklist

Unit of Analysis	10	• Description of the smallest unit that is being analyzed to assess intervention effects (e.g., individual, group, or community)		
		• If the unit of analysis differs from the unit of assignment, the analytical method used to account for this (e.g., adjusting the standard error estimates by the design effect or using multilevel analysis)		
Statistical Methods	11	• Statistical methods used to compare study groups for primary methods outcome(s), including complex methods of correlated data	✓	15-16
		• Statistical methods used for additional analyses, such as a subgroup analyses and adjusted analysis	✓	15-16
		Methods for imputing missing data, if used	✓	15-16
		Statistical software or programs used	✓	15-16
**Results**				
Participant flow	12	Flow of participants through each stage of the study: enrollment, assignment, allocation, and intervention exposure, follow-up, analysis (a diagram is strongly recommended)	✓	7, 8-13
		○ Enrollment: the numbers of participants screened for eligibility, found to be eligible or not eligible, declined to be enrolled, and enrolled in the study	✓	7, 8-13
		○ Assignment: the numbers of participants assigned to a study condition	✓	7, 8-13
		○ Allocation and intervention exposure: the number of participants assigned to each study condition and the number of participants who received each intervention	✓	7, 8-13
		○ Follow-up: the number of participants who completed the follow-up or did not complete the follow-up (i.e., lost to follow-up), by study condition	✓	7, 8-13
		○ Analysis: the number of participants included in or excluded from the main analysis, by study condition	✓	7, 8-13
		• Description of protocol deviations from study as planned, along with reasons		
Recruitment	13	Dates defining the periods of recruitment and follow-up	✓	6-7, 8-13
Baseline Data	14	Baseline demographic and clinical characteristics of participants in each study condition	✓	7, 8-14
		Baseline characteristics for each study condition relevant to specific disease prevention research		
		Baseline comparisons of those lost to follow-up and those retained, overall and by study condition	✓	8-14
		Comparison between study population at baseline and target population of interest	✓	8-13
Baseline equivalence	15	• Data on study group equivalence at baseline and statistical methods used to control for baseline differences	✓	8-13
Numbers analyzed	16	• Number of participants (denominator) included in each analysis for each study condition, particularly when the denominators change for different outcomes; statement of the results in absolute numbers when feasible	✓	8-13, 15-16
		• Indication of whether the analysis strategy was "intention to treat" or, if not, description of how non-compliers were treated in the analyses		

For peer review only - http://bmjopen.bmj.com/site/about/guidelines.xhtml

TREND Statement Checklist

Outcomes and estimation	17	• For each primary and secondary outcome, a summary of results for each estimation study condition, and the estimated effect size and a confidence interval to indicate the precision	✓	15-16
		• Inclusion of null and negative findings	✓	15-16
		Inclusion of results from testing pre-specified causal pathways through which the intervention was intended to operate, if any		
Ancillary analyses	18	• Summary of other analyses performed, including subgroup or restricted analyses, indicating which are pre-specified or exploratory	✓	15-16
Adverse events	19	• Summary of all important adverse events or unintended effects in each study condition (including summary measures, effect size estimates, and confidence intervals)		
**DISCUSSION**				
Interpretation	20	• Interpretation of the results, taking into account study hypotheses, sources of potential bias, imprecision of measures, multiplicative analyses, and other limitations or weaknesses of the study	✓	18-19
		Discussion of results taking into account the mechanism by which the intervention was intended to work (causal pathways) or alternative mechanisms or explanations		
		Discussion of the success of and barriers to implementing the intervention, fidelity of implementation	✓	18-19
		Discussion of research, programmatic, or policy implications	✓	18-19
Generalizability	21	• Generalizability (external validity) of the trial findings, taking into account the study population, the characteristics of the intervention, length of follow-up, incentives, compliance rates, specific sites/settings involved in the study, and other contextual issues		
Overall Evidence	22	• General interpretation of the results in the context of current evidence and current theory	✓	3-4

From: Des Jarlais, D. C., Lyles, C., Crepaz, N., & the Trend Group (2004). Improving the reporting quality of nonrandomized evaluations of behavioral and public health interventions: The TREND statement. *American Journal of Public Health*, 94, 361-366. For more information, visit: http://www.cdc.gov/trendstatement/

For peer review only
